# Enzyme-Activatable Fluorogenic Probes: Design Strategies, Biomedical Applications, and Future Perspectives

**DOI:** 10.1021/jacs.5c22017

**Published:** 2026-07-14

**Authors:** Yufan Fan, LeLe Liu, Hairong Zeng, Zeyu Wang, Xiaojun Peng, Zhuang Hu, Tony D. James, Guangbo Ge

**Affiliations:** † State Key Laboratory of Discovery and Utilization of Functional Components in Traditional Chinese Medicine, Shanghai Frontiers Science Center of TCM Chemical Biology, Institute of Interdisciplinary Integrative Medicine Research, 66322 Shanghai University of Traditional Chinese Medicine, Shanghai 201203, China; ‡ Key Laboratory of Xinjiang Phytomedicine Resource and Utilization, Ministry of Education, Pharmacy School of Shihezi University, Xinjiang 832003, China; § State Key Laboratory of Fine Chemicals, Dalian University of Technology, 2 Linggong Road, Dalian 116024, China; ∥ Ningbo Municipal Hospital of Traditional Chinese Medicine (TCM), Affiliated Hospital to Zhejiang Chinese Medical University, Ningbo 315000, China; ⊥ Department of Chemistry, 1555 University of Bath, Bath BA2 7AY, U.K.; ○ School of Chemistry and Chemical Engineering, Henan Normal University, Xinxiang 453007, China

## Abstract

Enzymes are pivotal regulators of cellular metabolism and organismal homeostasis, and their dysregulation is often associated with the onset and progression of disease. Therefore, accurate monitoring of the real activities of target enzymes is essential for deciphering biological mechanisms and gaining pathological insight. Through decades of iterative refinement, a diverse repertoire of enzyme-activatable fluorogenic probes (EAFPs) have been developed, enabling the capture of aberrant enzyme dynamics with high spatiotemporal resolution and multifunctional biosensing capabilities. In this context, we highlight the current state-of-the-art EAFPs, spanning fundamental design principles to proof-of-concept applications. First, the molecular engineering, sensing mechanisms, and design strategies of EAFPs are introduced. Next, a wide range of cutting-edge probes for imaging and sensing target enzyme(s) are presented, with emphasis on structural features, recognition mechanisms, and biomedical applications. Representative examples in biomarker imaging, disease diagnosis, drug screening, and therapeutic testing are highlighted to illustrate both the design principles and practical utility. Finally, the existing challenges and future trajectories for EAFPs in specific application scenarios are discussed. The insights presented here will inspire and accelerate the development of high-performance multifunctional EAFPs for both fundamental and translational research.

## Graphical abstract

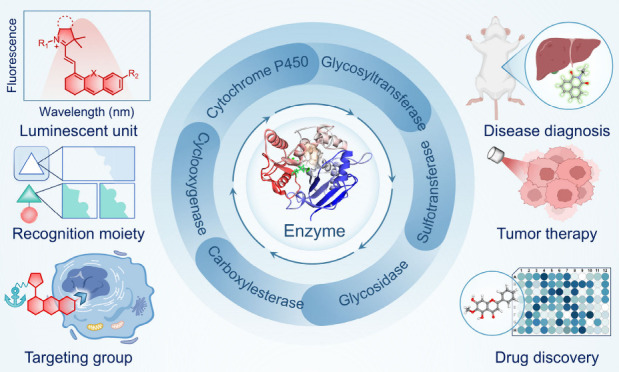

## 1 Introduction

Enzymes, as pivotal biocatalysts driving the metabolic networks of life, have emerged as crucial biomarkers and prognostic indicators in various pathological conditions.^1^ Their expression profiles and functional states can be modulated by multiple factors such as genetics, gender, pathology, and environmental influences. Unveiling enzyme dynamics within native microenvironments is vital for disease diagnosis and targeted therapy. Conventional proteomic techniques, such as Western blot and LC-MS/MS, acquire static protein abundance profiles but lack insight into the enzyme activity dynamics. These methods are constrained by labor-intensive protocols, low-throughput detection, and inherent deficiencies in spatiotemporal resolution.^2^ This technical bottleneck has spurred the evolution of enzyme-activatable fluorogenic probes (EAFPs). EAFPs remain optically silent in native states, yet trigger programmable signal amplification or wavelength shifts upon enzyme-mediated activation, including specific binding or chemical reactions.^3–5^ By transducing enzyme expression or activity into quantifiable photonic signals, EAFPs enable the noninvasive visualization of activity dynamics, expression levels, and spatial distributions across various biological hierarchies. This capability has opened transformative avenues for pathological diagnosis, intraoperative navigation, and drug screening.^6^

Over the past decade, remarkable advances have been achieved in the architectural design and activation paradigms of the EAFPs. Near-infrared (NIR) imaging platforms, synergized with photoacoustic or positron emission tomography (PET) imaging, facilitate the high-contrast tracking of target enzymes from subcellular compartments to whole organisms.^7–9^ This integration benefits from minimized autofluorescence, enhanced photon penetration, and an unparalleled imaging resolution. Regarding responsive modes, dual-lock activation systems that necessitate simultaneous dual-enzyme activation and/or microenvironmental triggers (e.g., acidic pH, redox state) set new standards for enhancing biosensor specificity.^10–12^ Proximity labeling techniques employ electrophilic intermediates to enable covalent anchoring of activated probes to target enzymes, effectively circumventing signal diffusion.^13,14^ Artificial intelligence (AI)-powered platforms also accelerate the development of high-performance probes by identifying high-affinity fragments and predicting probe-enzyme binding modes.^15^ Despite these considerable strides, critical challenges persist. There remains a lack of in-depth exploration of the relationship between enzyme active site features and EAFP metabolism along with broadly applicable optimization strategies, limiting the performance optimization and application expansion of EAFPs.

A deep understanding of the enzyme’s active site topology, catalytic mechanism, and substrate preferences is essential for rational probe design. These features collectively inform the selection of luminescent units and the structural design of the trigger group. Against this backdrop, this perspective systematically illustrates how these intrinsic biological profiles of enzymes govern the molecular engineering of EAFPs. Recent advances in state-of-the-art EAFPs over the past decade (2015–2025) are outlined, focusing on cutting-edge design strategies and transformative biomedical applications. Initially, the engineering concepts, optical mechanisms, and sensing modes that underpin multifunctional EAFPs are expounded to improve accuracy. These guidelines empower researchers to rationally manipulate the photophysical properties of purpose-built dyes based on enzyme architectures and substrate preference, thereby illuminating the dynamics of enzyme expression and activity. Then, representative EAFPs are deliberated in accordance with their target enzymes’ physiological roles, highlighting intelligent molecular designs, key structural features, recognition mechanisms, and detection performance. Furthermore, we showcase breakthrough applications of EAFPs in biomarker analysis, multimodal imaging, drug screening, and tumor theranostics. Finally, the challenges and future perspectives for the optimization of EAFP are also presented. Collectively, this perspective will guide the iterative optimization of EAFPs and accelerate their translation into biomedical research and clinical practice.

## 2 Molecular Engineering and Design Strategies of EAFPs

Conceptually, EAFPs are a class of intelligent molecular systems that synergize luminescent signaling with molecular recognition. To fulfill their diagnostic and imaging potential, ideal EAFPs must satisfy multidimensional performance criteria.^14,16,17^ First, the core fluorophore should possess a high extinction coefficient, a favorable quantum yield, and well-matched emission profiles (preferably in the NIR region). These photophysical properties are critical for maximizing the signal-to-noise ratio (SNR), tissue penetration depth, and long-term monitoring capability. Second, EAFPs should exhibit exceptional specificity and ultrahigh sensitivity toward the target enzyme, enabling the rapid and reliable conversion of enzymatic activity into discernible spectroscopic changes (e.g., wavelength shifts and intensity variations). Finally, the probe must demonstrate favorable biocompatibility, including low cytotoxicity, efficient membrane permeability, precise subcellular targeting, and an acceptable metabolic stability. This Perspective will delve into the molecular engineering principles and sensing modes of purpose-built dyes, with the goal of advancing their biosensing accuracy and translational potential.

### 2.1 Basic Building Blocks in EAFPs

From a modular standpoint, EAFPs can be deconstructed into three core components: a fluorophore, a recognition moiety (RM), and a linker. The fluorophore functions as the luminescent reporter responsible for illuminating the spatial distribution, expression levels, and real activity of enzymes (Figure 1A).^6^ Its intrinsic photophysical properties, such as absorption and emission profiles, photothermal conversion efficiency, and reactive oxygen species (ROS) yield, govern the practical applicability of EAFPs across various scenarios, including multiphoton imaging, image-guided surgery, photoacoustic (PA) imaging, photothermal therapy (PTT), and photodynamic therapy (PDT).^17^ Representative fluorescent dyes include coumarin, naphthalimide, BODIPY, rhodamine, and cyanine dyes (e.g., QCy7).^16,18,19^ The RM endows the sensor with target-recognition and signal-conversion capabilities. Upon enzymatic activation, the RM induces pronounced changes in the electronic configuration and optical properties of the probe, dictating its isozyme specificity, detection sensitivity, and response speed (Figure 1B). In some cases, a linker bridging the fluorophore and RM facilitates an efficient enzymatic reaction and enables robust signal amplification (Figure 1C).

**Figure 1:**
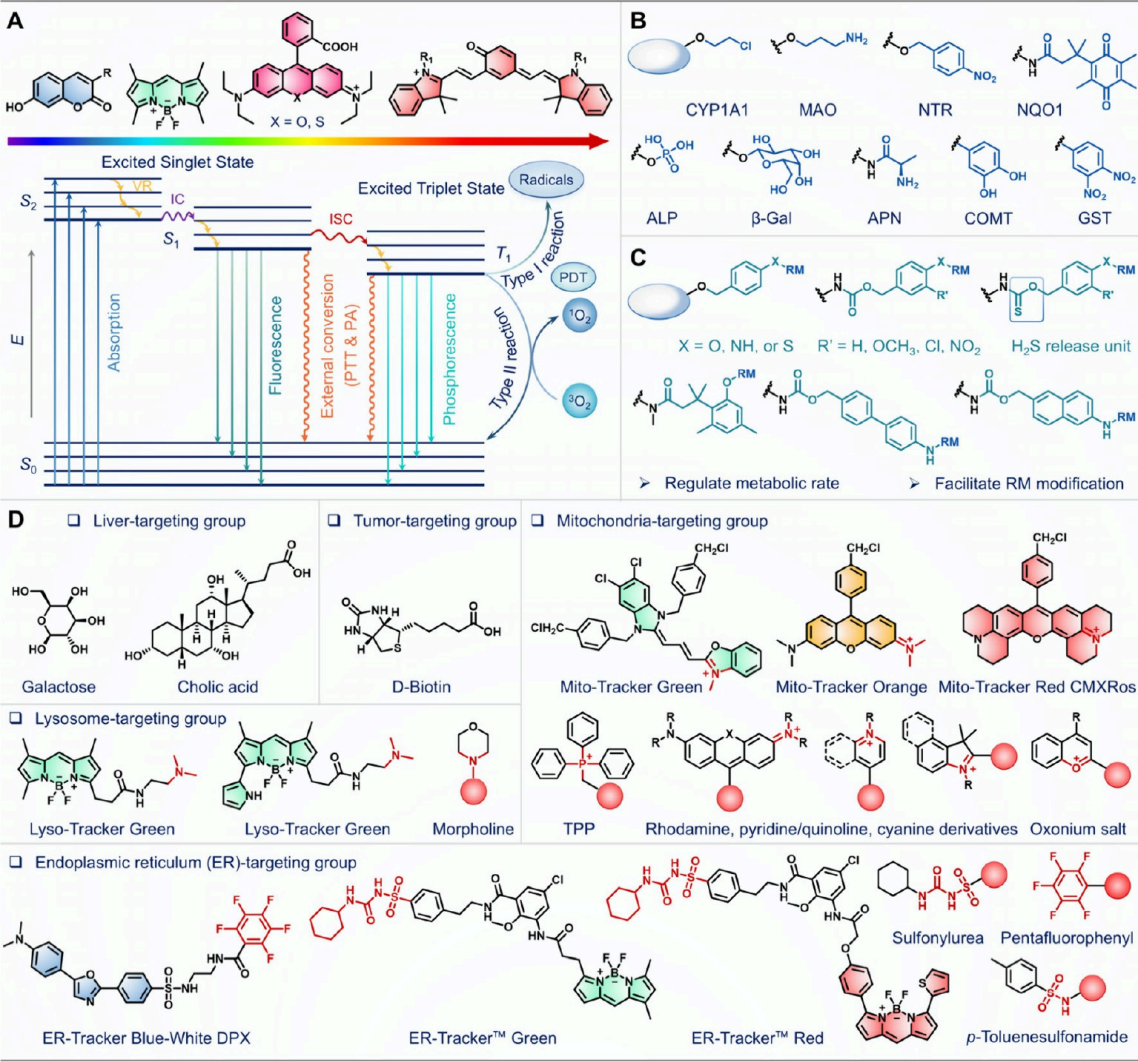
Molecular engineering and building blocks of EAFPs. (A) Jablonski energy level diagram of organic luminescent materials. VR, vibration relaxation; IC, internal conversion; ISC, intersystem crossing. (B) Specific RM for sensing various enzymes. (C) Typical self-immolative linkers. (D) Common organ-targeting and organelle-targeting groups.

Beyond these core architectures, specific targeting groups (TG) enable precise spatiotemporal navigation of EAFPs, significantly enhancing imaging fidelity for enzymes and related biological events (Figure 1D).^20–23^ Common targeting strategies exploit receptor–ligand recognition, microenvironmental stimuli (e.g., pH, lipophilicity, membrane potentials, or redox states), and passive targeting mechanisms (e.g., hepatic hemodynamics and Kupffer cell uptake).^24–27^ For instance, galactose-functionalized probes exploit the asialoglycoprotein receptor (ASGPR), which is highly expressed on hepatocytes, to achieve liver-selective accumulation.^28–30^ Organelle-specific localization can be accomplished with dedicated targeting groups. Driven by the negative mitochondrial membrane potential (MMP, −180 mV), lipophilic cationic dyes such as triphenylphosphonium (TPP) and quaternary ammonium salts often selectively label mitochondria.^22^ Lipophilic amines (e.g., morpholine) undergo protonation and are retained in acidic lysosomes (pH 4.5–5.5). For endoplasmic reticulum (ER) localization, *p*-toluenesulfonamide and sulfonylurea derivatives can bind specifically to sulfonylurea receptors on ER membranes.^31^ Additionally, introducing hydrophobic units (e.g., pentafluorophenyl, amphiphilic cationic groups, and long-chain alkyl groups) and optimizing overall lipophilicity (clog*P* > 3.4) of dyes can promote ER retention.

### 2.2 Sensing Types and Responsive Features of EAFPs

Based on the interaction mode between the RM and the target, EAFPs are broadly categorized into binding-based probes and activity-based probes. Binding-based probes rely on supramolecular recognition through noncovalent interactions rather than enzymatic reactions (Figure 2A). Functioning as reversible ligands, they enable real-time visualization of protein localization or expression levels as opposed to enzyme activity.^32^ A prevalent strategy involves conjugating a specific ligand (e.g., an inhibitor or functional peptide) to the fluorophore, establishing a specific lock-and-key system for protein localization.^33^ However, some “always-on” probes may necessitate multiple washing steps during bioimaging to minimize false-positive signals from unbound dyes.^34^ Environmentally sensitive dyes are highly suitable for tailoring such probes, especially twisted intramolecular charge transfer (TICT), aggregation-induced emission (AIE), and excited-state intramolecular proton transfer (ESIPT) dyes.^5,31^ Their fluorescence can be activated within the hydrophobic environment of the target enzyme’s active cavity by constraining conformational changes or stabilizing the keto–enol tautomerization state.

**Figure 2:**
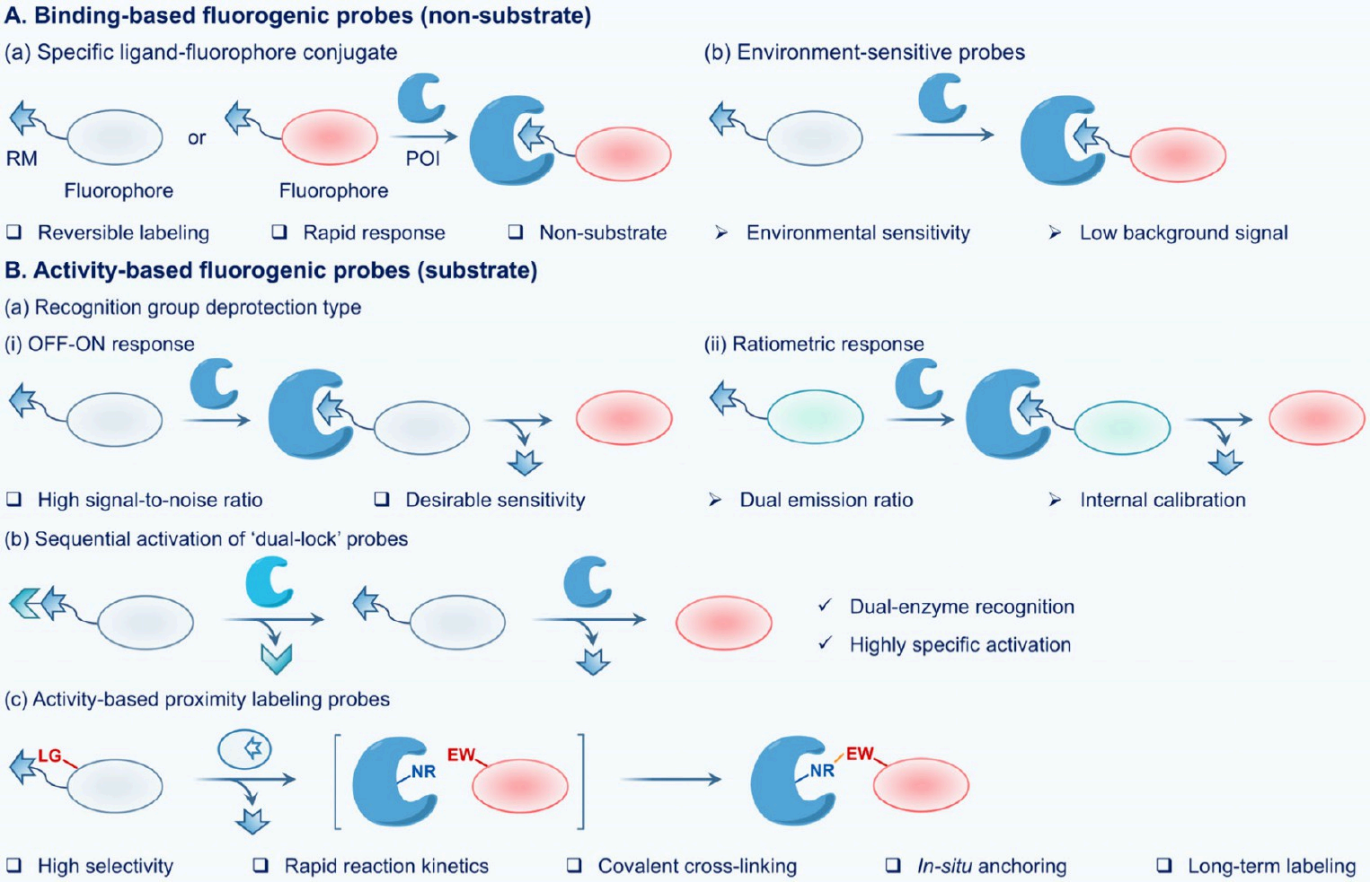
Sensing patterns and responsive characteristics of EAFPs.

Importantly, protein abundance does not always correlate with enzyme activity across spatiotemporal contexts. Therefore, activity-based probes have emerged as powerful tools for directly measuring enzyme activity, offering ultrahigh selectivity and point-of-care detection (Figure 2B).^35^ Common optical mechanisms, including intramolecular charge transfer (ICT), photoinduced electron transfer (PeT), AIE, and ESIPT, enable precise manipulation of the desired signal output. These probes operate through specific enzymatic reactions (e.g., oxidation or hydrolysis) that remove a masking group, triggering significant electronic rearrangement and generating an OFF–ON or ratiometric response.^36–38^ Such a deprotection strategy has been extensively used for oxidases (e.g., cytochrome P450 enzymes, monoamine oxidase, and nitroreductase) and hydrolases (e.g., carboxylesterases, phosphatases, and galactosidases). Beyond this deprotection mechanism, activity-based probe designs have evolved to encompass more sophisticated sensing modes. For instance, to improve diagnostic accuracy, intelligent AND logic probes incorporate two distinct recognition units in series, emitting fluorescence only upon dual-enzyme stimulation.^10,39^ A major limitation of conventional EAFPs is the spontaneous diffusion of fluorescent products, which compromises localization accuracy.^40,41^ To surmount this bottleneck, an enzyme-triggered proximity labeling strategy has been introduced for high-fidelity imaging of enzymes.^34,42^ Upon removal of the RM, the leaving groups (LG) of these probes generate highly reactive electrophilic warheads (EW) such as quinone methide (QM) intermediates that covalently cross-link with nucleophilic residues (NR) in proximal proteins, enabling permanent signal anchoring.^43,44^ Notably, the term “activity-based probes” is also used in the field of activity-based protein profiling (ABPP), where such probes typically comprise a reactive warhead (e.g., fluorophosphonate), a reporter group (biotin or fluorophore tag), and a linker.^45^ Unlike ABPP probes, which covalently label enzyme active sites for proteomic enrichment, the fluorogenic activity-based probes described here are usually silent until enzymatic activation and are designed for real-time imaging.

### 2.3 Design Workflow of High-Performance EAFPs

The development of specific EAFPs encounters substantial challenges arising from intricate enzymatic processes and interconnected metabolic networks. Rational probe design must be grounded in a thorough comprehension of the target enzyme’s biochemical traits, including catalytic profiles, substrate preference, subcellular localization, tissue distribution, and expression levels (Figure 3).^38^ The intended imaging scenarios guides the choice of fluorophore with appropriate optical window and photophysical properties, whereas metabolic profiles and substrate preferences dictate the structural features of both the fluorophore and RM. High-affinity ligands, including physiological substrates (e.g., arachidonic acid for cyclooxygenase-2), drug substrates (e.g., irinotecan for carboxylesterase 2A), and specific inhibitors (e.g., ritonavir for cytochrome P450 3A4), provide crucial structural insights for the modular assembly of EAFPs.^15,46,47^ Building on this foundation, contemporary EAFP design primarily follows two complementary paradigms: ligand-based probe design (LBPD), which exploits the functional fragments and structural features of known substrate or inhibitor scaffolds, and structure-based probe design (SBPD), which leverages three-dimensional structural information about the target enzyme.^48^

**Figure 3:**
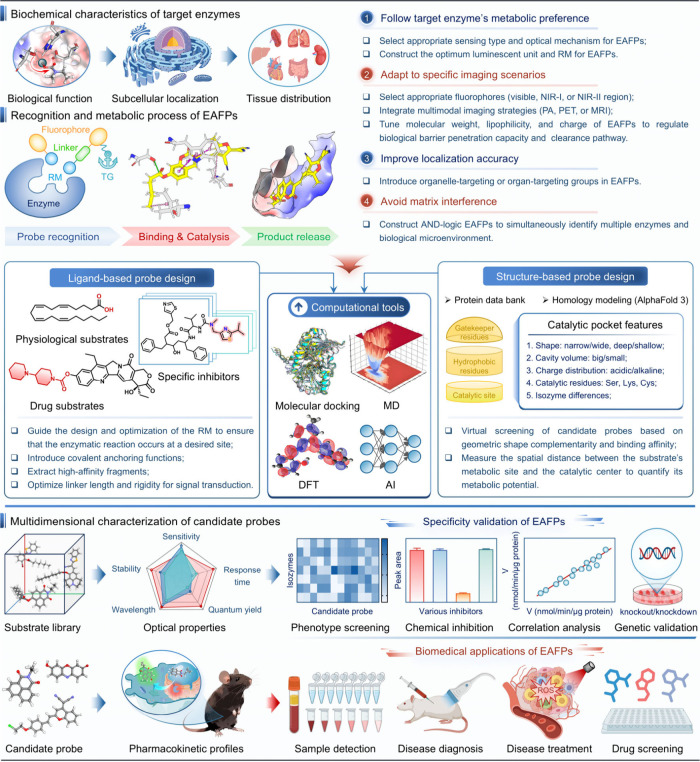
General workflow for rational construction and multidimensional characterization of high-performance EAFPs.

Substrate recognition and enzymatic catalysis are complex yet pivotal processes that govern the isozyme specificity and catalytic efficiency toward EAFPs. Even if an EAFP accesses the enzyme’s active cavity, an inappropriate spatial distance between its metabolic site and the enzyme’s catalytic center (e.g., serine, lysine) can substantially impede fluorescence activation.^38,49,50^ Hence, a comprehensive analysis of the active cavity’s topological architectures (e.g., shape, depth, and volume), local hydrophobicity, and structural variations among related isozymes is essential, a core tenet of SBPD.^51^ For those enzymes with narrow and deep active cavities, incorporating a self-immolative linker often represents an effective strategy to mitigate steric hindrance from bulky fluorophores while optimizing spatial orientation toward catalytic residues.^49^ Computational methods now play an indispensable role in accelerating both paradigms. Common techniques, including homology modeling (e.g., AlphaFold), virtual screening, 3D similarity searches, thermodynamic computations, and AI, enable parallel optimization of the fluorophore, RM, and linker by predicting probe-enzyme interactions, structure–activity relationships, and metabolic feasibility.^49,50,52^

Following computational design, a diversity-oriented substrate library is constructed and subjected to enzymatic phenotyping to establish structure-selectivity relationships.^3^ The rapid identification of lead probes with favorable optical performance, desirable specificity, and high turnover demands rigorous validation encompassing optical characterization and methodological testing. Systematic profiling of spectroscopic properties includes absorption/emission spectra, quantum yield, photostability, and detection sensitivity. The specificity of EAFPs is first examined using recombinant isozymes to determine kinetic parameters (*V*_max_/ *K*_m_) and selectivity ratios. Moving beyond purified enzyme models, chemical inhibition assays confirm EAFP specificity in complex biological samples by measuring reduced product formation after preincubation with selective inhibitors in cell lysates or living cells. Genetic manipulation (e.g., CRISPR-Cas9-mediated knockout or siRNA knockdown) offers a definitive strategy.^49,53^ In parallel, competition assays with native substrates can help evaluate whether the probe occupies the same active site as the endogenous substrate, thereby reinforcing functional relevance. Correlation analysis between the metabolic rates of EAFPs and those of specific drug or physiological substrates in biological matrices such as human liver microsomes (HLM) allows inference of the target enzyme’s contribution to EAFP metabolism.^15,54^ For *in vivo* studies, imaging of wild-type versus knockout animals, biodistribution assessment, and evaluation of off-target organ activation are critical to distinguish genuine enzyme activity from nonspecific accumulation or metabolism.^55^

For *in vivo* imaging, the physicochemical and pharmacokinetic properties of EAFPs, including solubility, lipophilicity, metabolic stability, plasma protein binding, clearance kinetics, tissue distribution, and safety, critically determine imaging performance in addition to the photophysical characteristics.^56,57^ Although most EAFPs are administered intravenously or locally and thus bypass the first-pass effects, their inherent structural features often impose significant pharmacokinetic liabilities. Specifically, EAFPs with extended π-conjugation and high hydrophobicity usually exhibit poor aqueous solubility and diminished metabolic stability, leading to predominant hepatic uptake.^58^ Conversely, EAFPs with good water solubility (e.g., PEGylated probes) or additional positive charges tend to exhibit rapid renal clearance.^59,60^ The clearance route and metabolic half-life of EAFPs synergistically influence imaging specificity, SNR, and compatibility with clinical workflows. Beyond pharmacokinetics, safety considerations also warrant attention. Certain EAFPs contain structural alerts, i.e., structural units or functional groups that may either directly react with cellular components or undergo metabolic activation to generate reactive intermediates. Such intermediates can covalently modify cellular proteins or DNA, potentially posing toxicity, immunogenicity, or other adverse biological effects. Representative examples include quinone moieties, Michael acceptors, nitro groups, and thiophene rings.^61^ These translational constraints, spanning biological stability, clearance behavior, dosing schedule, and safety, represent recurring hurdles that often preclude promising probes from advancing beyond proof-of-concept studies.

Collectively, the above criteria form a practical and rigorous roadmap for validating EAFP specificity and biological compatibility, ensuring that imaging results accurately reflect the target enzyme activity. This stringent validation pipeline guarantees that the optimized EAFPs can reliably monitor enzyme activity in complex biological environments from cellular systems to *in vivo* models, enabling multidimensional functional imaging across multiple biological scales.^62,63^ Ultimately, high-performance probes are integrated into automated detection platforms, facilitating investigations of cellular metabolism, organ function assessment, pathological mechanism elucidation, and large-scale drug screening.

## 3 Recent Advances of EAFPs

Enzymes constitute a fundamental biochemical defense system that protects organisms from diverse external xenobiotics and toxicants. Phase I drug-metabolizing enzymes, such as cytochrome P450 (CYPs), introduce polar groups into lipophilic compounds through oxidation, reduction, or hydrolysis.^1^ Subsequently, phase II drug-metabolizing enzymes, such as uridine diphosphate glucuronosyltransferases (UGTs), further enhance the hydrophilicity of these compounds via conjugation reactions like glucuronidation, facilitating biliary or renal excretion. Clarifying the functional kinetics and regulatory networks of these enzymes has emerged as a key research direction, which is indispensable for deciphering the biomarker significance and guiding clinical decision making. Against this backdrop, this Perspective systematically discusses representative EAFPs, with a focus on rational molecular design, underlying sensing mechanisms, and emerging biomedical applications (Figure 4). The sensing performance of the representative EAFPs is summarized in Table 1.

**Figure 4:**
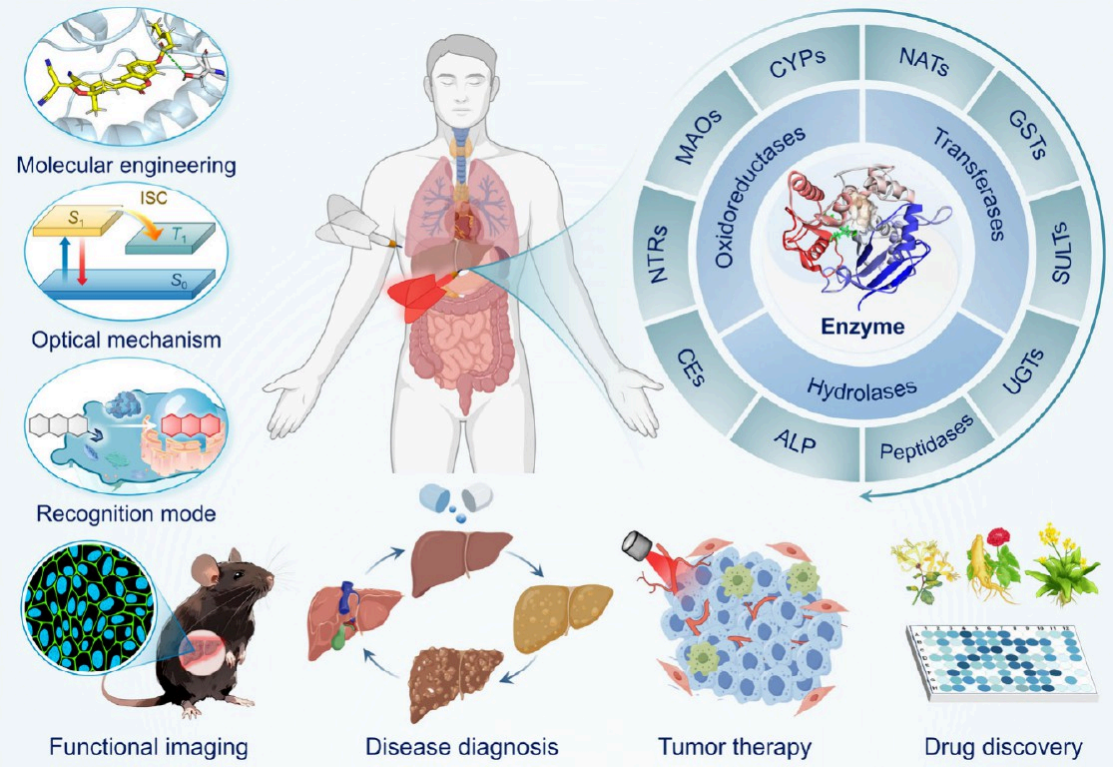
Overview of molecular engineering and biomedical applications of EAFPs.

**Table 1:** Sensing Performance of Representative EAFPs

probe	enzyme	mechanism	λ_ex_/λ_em_ (nm)	*K*_m_ (μM)	SNR (fold)	biological samples	ref
1	CYP1A1	ICT	550/672	–	18.6	long-term tracking of cancer cell metastasis and invasion	^14^
2	CYP2D6	ICT	520/558	4.90	–	imaging of CYP2D6 activity in tumor cells, primary neural cells, and brain slices	^64^
						inhibitor screening	
3	CYP2J2	ICT	656/718	4.20	–	imaging of CYP2J2 activity in tumor cells, angiogenesis, tumor-bearing nude mice	^49^
4	CYP3A4	ICT	450/558	59.80	–	imaging of CYP3A4 activity in primary hepatocytes and living zebrafish	^65^
5	CYP3A4	ICT	450/555	0.36	–	sensing CYP3A4 activity in HLM	^66^
						imaging of CYP3A4 activity in Hep3B2 cells and mouse liver slice	
						screening and *in vivo* evaluation of CYP3A4 inhibitors	
6	CYP3A4	ICT	450/555	11.42	154.2	imaging of CYP3A4 activity in HLM samples, living cells, liver, and tumor-xenografted mice	^15^
						screening and *in vivo* evaluation of CYP3A4 inhibitors	
7	CYP3A4	ICT	450/555	4.27	125	imaging of CYP3A4 activity in HLM samples, living cells, and liver	^54^
						multidimensional inhibitor assessment	
8	MAO-A	TICT	430/600	–	–	MAO-A imaging in cells, human glioma and paracancerous tissues	^67^
9	MAO-B	ICT	680/715	–	–	MAO-B imaging in liver fibrosis cells and mice models	^68^
10	COX-2	PeT	457/547	–	28	imaging of COX-2 expression in tumor cells and tissues	^32^
						specific localization of Golgi apparatus of cancer cells	
11	COX-2	–	380/398	–	–	imaging the endogenous H_2_O_2_ in COX-2 overexpressing tumor cells	^69^
			380/554				
12	COX-2	ICT	540/590	22	–	imaging of COX-2 activity in living macrophage cells	^46^
13	NTR	PeT	730/910	–	6.4	imaging of NTR in subcutaneous pancreatic cancer tumor models	^70^
						NIR-II image-guided surgical resection of multimicrotumors	
14	NTR	ICT	670/750	–	11	NIR-PA dual-modal imaging of NTR for quantitative evaluation of PTT efficacy *in vivo*	^71^
			770/850				
15	NQO1	ICT	580/695	–	245	imaging of NQO1 activity in HT-29 cells and tumor-bearing mice	^72^
16	NQO1	ICT	650/705	–	–	induce pancreatic cancer cell death	^73^
						*ex vivo* imaging of pancreatic cancer sections and solid tumors	
17	AzoR	ICT	430/540	–	–	imaging of AzoR activity in the mitochondria of living cells	^74^
18	AzoR	ICT	680/716	–	14	selective activation by tumor hypoxia to release chemotherapeutic drugs and photosensitizers	^75^
19	CES1A	ICT	530/595	2.10	–	monitor CES1A activity in living cells, tissue slices, organs, and zebrafish	^76^
20	CES1A	ICT	600/690	11.22	–	imaging of CES1A under ER stress and acute liver injury models	^77^
21	CES1A	ICT	600/670	4.20	–	imaging of endogenous CES1A activity in living cells	^78^
						evaluate the inhibitory effects of pesticides against CES1A	
22	CES2A	ICT	354/452	8.58	80	CES2A activity in HLM samples	^79^
			430/542			imaging of CES2A in living cells and mouse liver slices	
23	CES2A	ICT	600/662	1.92	57	visualization of CES2A in cells, various tissues, and *in vivo*	^80^
24	CES2A	ICT	570/670	25.43	61.3	imaging of CES2A in living cells, mouse liver slices, and tumor-xenograft mice	^50^
						high-throughput screening of CES2A inhibitors	
25	MAGL	–	489/502	–	–	specific labeling of MAGL in living cells, hippocampal neuron cultures, and brain organoids	^55^
26	ALP	ESIPT	410/550	43.30	100	visualization of ALP activity in osteosarcoma cells and tissue	^81^
27	ALP	ICT	680/710	23.70	–	*in situ* labeling of the ALP-positive HeLa cell membrane	^82^
						discriminate HeLa tumor foci from the normal tissues *in vivo*	
28	β-Gal	ICT	685/717	–	42	visualization of β-gal activity in tumor cells and mice	^62^
						precise removal of tumor tissue during surgical procedures in mice	
29	β-Gal	ICT	690/710	48.04	6.5	NIR-PA dual-modal imaging of tumor senescence	^83^
30	β-Gal	ICT	640/740	–	–	identify senescence in living cells and tumor-bearing mice	^84^
31	FAAH	ICT	550/592	1.84	–	FAAH imaging in living cells and inhibitor screening	^85^
32	vanin-1	ICT	665/710	6.86	85	imaging of Vanin-1 levels in inflammation models	^86^
33	PGA	PeT	770/787	4.24	–	promote bacterial infection treatment and wound healing by PTT and PDT	^87^
34	APN	ICT	686/708	–	15.8	high-precision imaging of tumor boundaries	^88^
35	FAPα	ICT	570/682	–	12.1	distinguish invasive tumor from benign lesions	^89^
36	cathepsin B	ICT	808/988	–	13.5	identification of metastatic lung lesions in unshaved mice	^90^
37	UGT1A9	ICT	465/608	0.57	50	evaluate the effects of pollutants against UGT1A9	^91^
						imaging of UGT1A9 in HepRG1A9 cells	
38	UGTs	ICT	360/450	–	–	high-throughput screening of UGT inhibitors	^92^
39	UGT1A1	ICT	450/564	126.70	155	measure UGT1A1 activity in liver preparations	^93^
			362/450			screening of UGT1A1 modulators	
						UGT1A1 imaging in HepG2 cells	
40	UGT1A1	PeT	370/520	0.70	20	high-throughput screening of UGT1A1 modulators	^94^
						interindividual variability of UGT1A1 activity	
41	UGT1A1	PeT	350/485	10.30	80	discovery of UGT1A1 inhibitors and activators in licorice	^95^
						imaging of UGT1A1 activity in HepG2 cells	
42	UGT1A10	PeT	340/455	32.80	15	imaging of UGT1A10 in cells, rat tissues, and zebrafish	^96^
						UGT1A10 inhibitor screening	
43	UGT1A1	–	670/720	–	–	imaging of endogenous UGT1A1 in HepG2 cells and animals	^53^
						monitor the bile excretion function	
						UGT1A1 inhibitor screening	
44	SULT1E1	PeT	360/510	4.93	69	sensing SULT1E1 activity in hepatocellular carcinoma specimens and live organs	^3^
						SULT1E1 inhibitor screening	
45	SULT1E1	PeT	360/510	1.67	212	sensing SULT1E1 activity in cellular specimens and liver preparations	^97^
						SULT1E1 inhibitor screening	
46	COMT	PeT	390/510	0.79	149	characterization of COMT inhibitors	^98^
						sensing COMT activity in individual erythrocyte samples	
						imaging of COMT in U87-MG cells and rat brain tissue slices	
47	NAT2	PeT	550/580	90.42	–	imaging of NAT2 in different bacterial strains	^99^
						NAT2 inhibitor screening	
48	NAT2	PeT	498/512	7.68	–	imaging of NAT2 in living bacteria	^100^
						NAT2 inhibitor screening	
49	NAT2	TICT	500/700	–	56	imaging of NAT2 in living cells and tissue homogenates	^101^
50	GSTs	ICT	730/810	21.34	–	imaging of elevated GSTs in the pulmonary fibrosis cells and mice models	^102^
						imaging of GSTs in the sample of IPF patient	
51	GSTs	ICT	630/686	–	6.7	imaging of overexpressed GSTs in cholestatic mice models	^103^
						sensing GSTs levels in the serum samples of the ICP patients	

### 3.1 Oxidoreductases

Oxidoreductases have emerged as pivotal biomarkers and therapeutic targets because of their intimate involvement in redox homeostasis, metabolic detoxification, and redox signaling. This section outlines the development of oxidoreductase-activatable probes, including CYPs, monoamine oxidases (MAOs), cyclooxygenases (COXs), nitroreductases (NTRs), NAD­(P)­H:quinone oxidoreductase-1 (NQO1), and azoreductase (AzoR).

#### 3.1.1 Cytochrome P450 Enzymes

CYPs constitute a superfamily of heme-containing proteins that mediate the oxidative metabolism of over 75% of clinical drugs. In humans, 57 CYP genes have been identified and classified into 18 families and 44 subfamilies. To avoid potential drug–drug interaction (DDI) risks, the U.S. Food and Drug Administration (FDA) requires drug development pipelines to assess whether candidate drugs cause reversible or time-dependent inhibition (TDI) of key CYP isoforms, including CYP1A2, CYP2B6, CYP2C8, CYP2C9, CYP2C19, CYP2D6, and CYP3A.^104^ CYPs typically catalyze O-dealkylation and aromatic hydroxylation of hydrophobic polyaromatic substrates, introducing polar groups (e.g., −OH) to increase hydrophilicity (Figure 5A). A critical design consideration is the strategic management of metabolic soft spots (i.e., functional groups or atoms prone to enzymatic metabolism). Many fluorophores contain multiple potential metabolic sites, leading to diverse metabolites that complicate activity quantification. Therefore, it is essential to deactivate alternative metabolic sites via strategic fluorination, steric blocking, and bioisosterism replacement.^4,66^ After identifying a suitable fluorogenic scaffold, a tailored recognition moiety can be introduced to direct metabolism to a desired site, acting as a fluorescence switch. Computational methods further aid in predicting potential metabolic sites.

**Figure 5:**
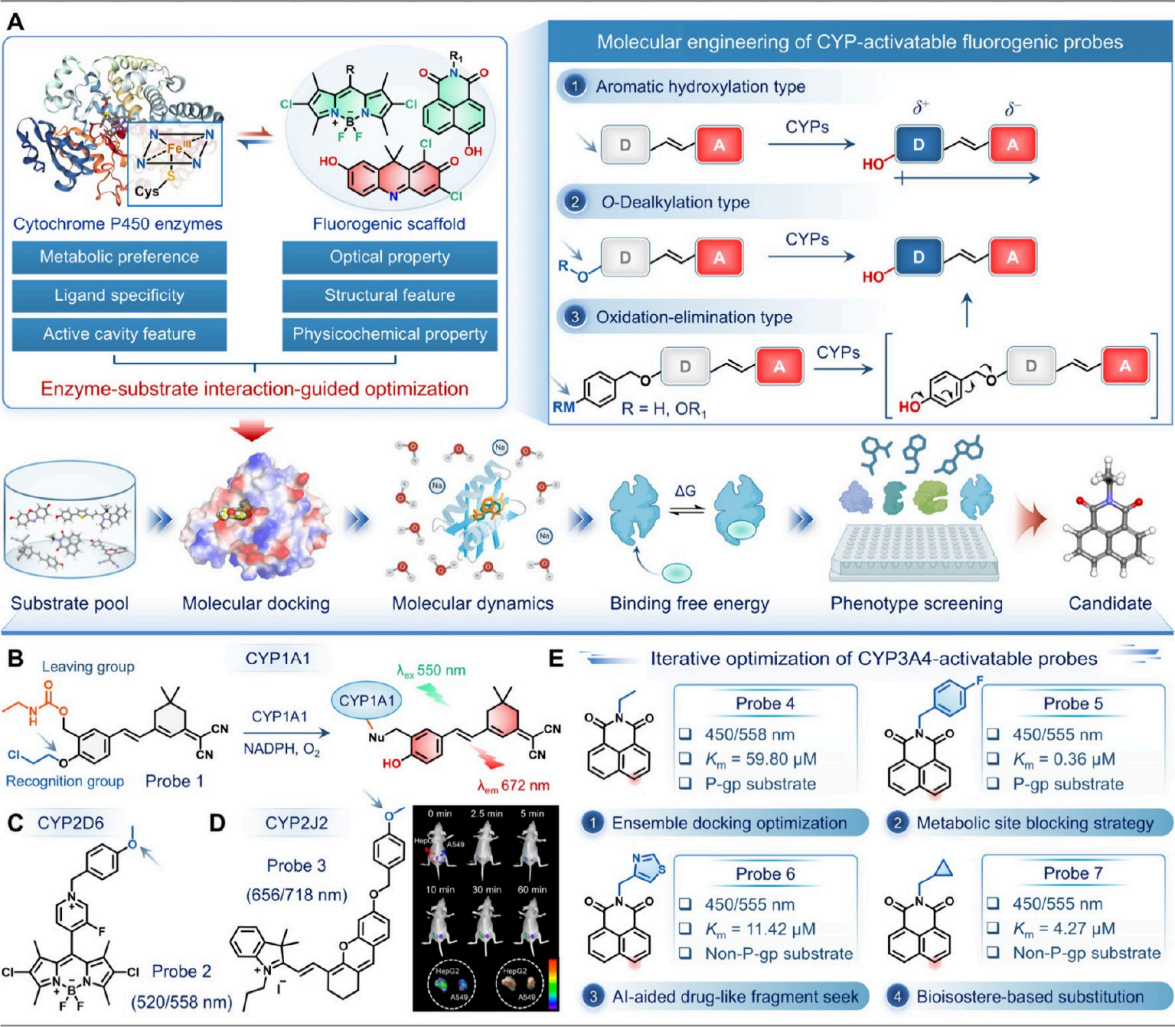
(A) Molecular construction, sensing modes, and optimization workflow of CYP-activatable probes. (B–E) Representative CYP probes and their detection wavelength (λ_ex_/λ_em_), including CYP1A1 (B), CYP2D6 (C), CYP2J2 (D), and CYP3A4 (E). Reproduced from ref 49. Copyright 2018 American Chemical Society.

Generally, a chloroethyl group serves as a specific RM for constructing CYP1A1 probes.^36,105,106^ Building on this design, probe 1 achieved specific recognition and long-term labeling of CYP1A1 through an enzyme-activated self-immobilization cascade (Figure 5B).^14^ Upon CYP1A1-mediated oxidative cleavage, the probe generated a highly electrophilic QM intermediate that covalently bound to CYP1A1, enabling long-term tracking of cancer cell metastasis and invasion in living mice for up to 4 days. In a separate approach, a tailored pyridine-BODIPY hybrid probe was developed to elucidate the pathological functions of CYP2D6 (Figure 5C).^64^ Screening a diverse substrate library identified an optimal fluorophore, followed by halogen substitutions to enhance binding affinity and catalytic efficiency. Probe 2 enabled real-time imaging of CYP2D6 activity in cancer cells, tissue slices, and neurological models, and facilitated the discovery of mechanism-based inactivators (e.g., isoimperatorin). For CYP2J2, which features a constricted catalytic cavity near the heme group that limits bulky substrates access, probe 3 was constructed by incorporating a self-immolative linker (Figure 5D).^49^ This design truncated the spatial distance and reduced steric hindrance between the metabolic site and the catalytic heme iron. Following CYP2J2-catalyzed O-demethylation and subsequent 1,6-elimination, the probe visualized CYP2J2 activity in diverse biological processes, including tumor migration, angiogenesis, and hematological malignancies.

The development of CYP3A4 probes exemplifies iterative optimization (Figure 5E). Initial efforts employed a two-dimensional strategy integrating ensemble docking-based two-photon fluorophore screening with N-substituent optimization, yielding a two-photon probe for CYP3A4 visualization in cells and zebrafish.^65^ However, this first-generation probe suffered from limited affinity (*K*_m_ = 59.8 μM) and poor membrane permeability. A second-generation probe improved affinity by introducing a benzene ring at the N site and a fluorine atom at the *para* position, effectively blocking alternative metabolic sites and directing metabolism to the C-4 position of naphthalimide.^66^ Despite improved imaging resolution and tissue penetration, both probes were identified as P-glycoprotein (P-gp) substrates, severely limiting their intracellular accumulation. To overcome this limitation, an AI-driven strategy was performed to identify drug-like fragments with high CYP3A4 affinity and oral bioavailability from an oral CYP3A4 inhibitor library.^15^ The first orally available probe 6 was developed by fusing a ritonavir-derived thiazole moiety with a naphthalimide scaffold, achieving exceptional ER localization and oral bioavailability.^15^ Concurrently, probe 7 was systematically optimized through bioisostere fragment growth, drug-likeness filtering, ensemble docking, and biochemical analysis.^54^ Probes 6 and 7 effectively circumvented P-gp-mediated efflux, demonstrating superior specificity, ultrahigh sensitivity, and improved membrane permeability. These optimized probes served as robust substitutes for physiological substrates (e.g., testosterone), facilitating functional CYP3A4 imaging across living systems, high-throughput inhibitor screening, and multidimensional pharmacological characterization. The iterative optimization of CYP probes reveals a clear paradigm: from early single-enzyme detection toward enzyme-activated self-immobilization for long-term tracking, and from empirical screening to structure-guided and AI-accelerated design. These advances underscore the importance of integrating metabolic soft spot management, linker engineering, and computational prediction in achieving isoform-selective CYP probes.

#### 3.1.2 Monoamine Oxidases

MAOs are flavin adenine dinucleotide (FAD)-containing enzymes anchored to the mitochondrial outer membrane, where they catalyze the oxidative deamination of biogenic amines to generate the corresponding aldehydes or ketones (Figure 6A). This function is central for maintaining amine homeostasis and regulating neurotransmitter levels. The MAO family comprises two isoforms, MAO-A and MAO-B, which share approximately 70% sequence identity but exhibit distinct substrate preferences.^107^ MAO-A mainly metabolizes larger neurotransmitters such as serotonin and norepinephrine, whereas MAO-B preferentially processes smaller substrates, including benzylamine and phenylethylamine. Selective recognition of these two isoenzymes remains challenging due to their high structural similarity. To address this issue, a computer-assisted screening was used to optimize a binding-based *N,N*-dimethyl-naphthylamine probe 8 for MAO-A (Figure 6B).^67^ The 3-amino-propyl substitution on the *N*-pyridine moiety could enhance compatibility with the MAO-A microenvironment and strengthen mitochondrial localization, enabling rapid (within 20 s) and selective MAO-A imaging in living cells and human glioma tissues. For MAO-B, a NIR probe 9 exhibited a marked fluorescence increase upon enzymatic activation and effectively distinguished fibrotic from healthy states in cellular and animal models (Figure 6C).^68^ The development of MAO-selective probes highlights the value of computational screening for identifying isoform-specific RM and the importance of tailoring probe architecture to the unique microenvironment of each isoform.

**Figure 6:**
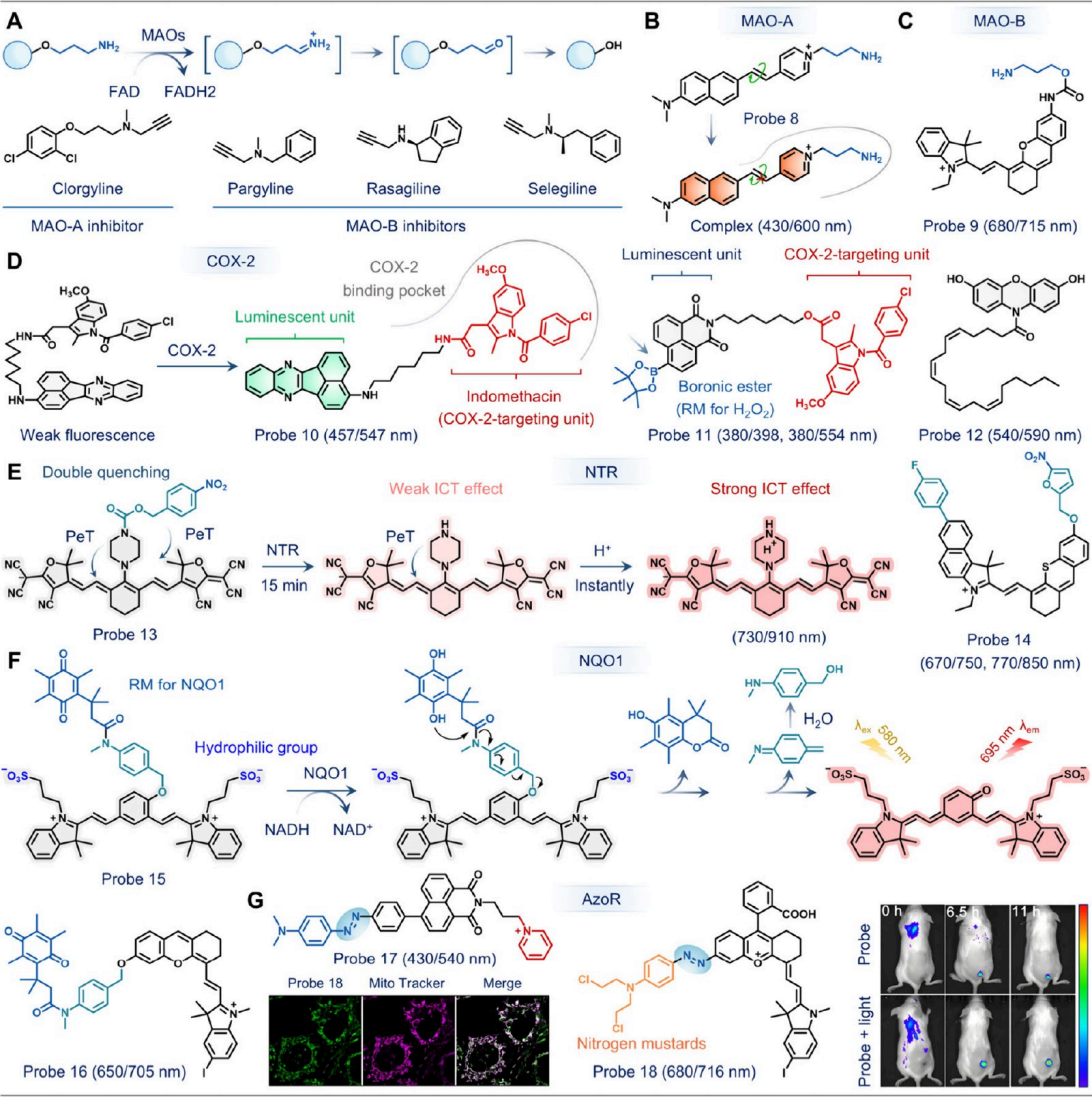
(A) Conventional probe design strategies for MAOs. (B, C) MAO fluorogenic probes 8 and 9. (D) COX-2 fluorogenic probes 10–12. (E) NTR fluorogenic probes 13 and 14. (F) NQO1 fluorogenic probes 15 and 16. (G) AzoR fluorogenic probes 17 and 18. (left) Mitochondrial colocalization analysis of probe 17. Reproduced from ref 74. Copyright 2024 American Chemical Society. (right) *In vivo* imaging of AzoR in BALB/c mice bearing 4T1 tumors after intravenous injection with probe 18. Reproduced with permission from ref 75. Copyright 2022 Wiley-VCH.

#### 3.1.3 Cyclooxygenases

Cyclooxygenases (COXs), comprising two primary isoforms (COX-1 and COX-2), catalyze the conversion of arachidonic acid to prostaglandin. While COX-1 is constitutively expressed in most normal tissues, COX-2 is markedly upregulated under pathological conditions, such as inflammation and cancer. The binding-based probe 10 exists in a folded state in aqueous solution, where fluorescence was quenched via a PeT process between an acenaphtho­[1,2-*b*]­quinoxaline fluorophore and an indomethacin moiety (Figure 6D).^32^ Upon binding to COX-2, the probe undergoes a conformational transition to an extended state, suppressing PeT and triggering intense fluorescence, enabling rapid discrimination between cancer and normal cells. Expanding on this strategy, a series of analogous probes have been developed by conjugating various COX-2 inhibitors (e.g., indomethacin, celecoxib) with fluorophores (e.g., Nile blue, naphthalimide).^33,108^ Recent advances included the development of dual-locked sensors (e.g., probe 11) capable of concurrent response to COX-2 and tumor microenvironment biomarkers (e.g., elevated H_2_O_2_ levels), allowing precise imaging of endogenous H_2_O_2_ in COX-2-overexpressing cancer cells.^69^ In parallel, activity-based probe 12 was designed using arachidonic acid linked to 3,7-dihydroxyphenoxazine (the reduced form of resorufin), permitting direct visualization of COX-2 activity in living macrophages. Two complementary design strategies were used for the development of COX-2 fluorogenic probes, including binding-based probes leveraging conformational changes upon target engagement and activity-based probes that directly report enzymatic turnover. The evolution toward dual-locked sensors further highlights the value of integrating multiple microenvironmental cues to enhance the specificity and achieve precision imaging in complex pathological settings.

#### 3.1.4 Nitroreductases

NTRs are flavoenzymes that catalyze the reduction of nitro groups to amines using nicotinamide adenine dinucleotide (NADH) or nicotinamide adenine dinucleotide phosphate (NADPH) as cofactors. NTR probes often incorporate a *p*-nitrobenzyl moiety as a fluorescence quencher. Upon NTR-mediated reduction, self-immolation releases the native fluorophore, generating a turn-on signal. A tandem-locked NIR-II probe 13 was engineered to respond to tumor hypoxia (NTRs) and the acidic tumor microenvironment for high-contrast imaging across diverse cancer types (Figure 6E).^70^ Its cascade activation mode minimized false positives and clearly delineated tumor boundaries, achieving a tumor-to-normal tissue (T/N) ratio of 7.8 and facilitating submillimeter precision for microtumor resection in preclinical models. In a parallel theranostic approach, probe 14 simultaneously generated NIR fluorescence, photoacoustic signals, and photothermal effects upon NTR activation, enabling real-time quantitative assessment of tumor cell death during photothermal therapy, a novel paradigm for image-guided precision treatment.^71^ NTR-responsive probes are evolving from simple hypoxia sensors into sophisticated multifunctional platforms for cancer diagnosis, image-guided surgery, and therapeutic monitoring, demonstrating the value of integrating multiple activation locks and multimodal imaging capabilities into a single probe architecture.

#### 3.1.5 NAD­(P)­H:Quinone Oxidoreductase-1

NQO1 is a two-electron reductase that utilizes NAD­(P)H to catalyze the reduction of quinones to hydroquinones, playing pivotal roles in maintaining cellular redox homeostasis and detoxifying quinone-based xenobiotics. NQO1 is abnormally upregulated in various solid tumors, and its aberrant regulation has also been implicated in the pathogenesis of neurodegenerative disorders.^109^ Utilizing the *para*-benzoquinone group as the NQO-1 specific RM, probe 15 displayed an ultrahigh SNR (∼245-fold) upon NQO1 addition, revealing elevated NQO1 activity in HT-29 cells and tumor-bearing mice (Figure 6F).^72^ The introduction of hydrophilic sulfonates improved the probe’s solubility and turnover rate. Similarly, hemicyanine-based probe 16 displays intensive NIR fluorescence following NQO1-catalyzed reduction and a self-immolative cleavage cascade.^73^ Probe 16 could achieve intraoperative pathological diagnosis of pancreatic cancer sections, whose fluorescent product was specifically enriched in mitochondria and lysosomes and exhibited effective chemotherapeutic-like effects to induce pancreatic cancer cell death via the cell pyroptosis pathway. Future development of high-fidelity NQO1 probes should focus on integrating NIR fluorophores to achieve deeper tissue penetration and a higher SNR.

#### 3.1.6 Azoreductase

AzoR is commonly upregulated in the hypoxic microenvironments of solid tumors, making it a compelling target for tumor diagnosis and therapy. AzoR-responsive probes typically function as quencher-release systems. Upon AzoR-mediated reduction, the azo bond is cleaved to generate aniline derivatives, thereby restoring fluorescence.^110^ A mitochondria-targeting platform was built on an *N*-pyridinium-4-amino-1,8-naphthalimide scaffold for visualizing mitochondrial AzoR activity in living cells (Figure 6G).^74^ Probe 17 revealed varying levels of this enzymatic activity across different cell lines, providing the first evidence of oxygen-insensitive intramitochondrial AzoR activity. In a theranostic approach, probe 18 was designed by linking a nitrogen mustard chemotherapeutic agent to a mitochondria-targeting NIR photosensitizer via a hypoxia-sensitive azo bond.^75^ Upon entering hypoxic tumor regions, the azo bond was cleaved by AzoR to release the active drug and photosensitizer. The release process could be monitored in real-time via fluorescence and photoacoustic imaging. Notably, oxygen consumption during PDT further exacerbated tumor hypoxia, creating a positive feedback loop that enhanced prodrug activation, drug release, and synergistic anticancer effects. The liberated photosensitizer accumulated in mitochondria, inducing tumor cell apoptosis and achieving improved therapeutic outcomes *in vivo* with minimal systemic toxicity. This work provided a novel strategy for enhancing hypoxia-activated prodrug release through PDT while enabling real-time imaging feedback. Looking forward, the natural metabolism of AzoR inspires the development of novel fluorescence-based prodrugs. Integrating AzoR activation with bioorthogonal chemistries will be crucial for advancing this class of stimuli-responsive agents in applications such as fluorescence-guided surgery and precision theranostics.

### 3.2 Hydrolases

Hydrolases are responsible for catalyzing the hydrolysis of ester-, amide-, glycoside-, or peptide-bond-bearing substrates. A general strategy for hydrolase-activatable probes involves masking a fluorophore’s auxochrome (e.g., −OH, −NH_2_) with an enzyme-specific moiety (e.g., ester groups for esterases) to suppress fluorescence, which is restored upon enzyme-mediated hydrolysis (Figure 7). The strategic integration of self-immolative spacers or covalent labeling enables the precise tuning of selectivity and sensitivity. This rational design paradigm has yielded a powerful toolkit for real-time visualization of hydrolases across numerous biological systems, driving development in disease diagnosis and drug discovery.

**Figure 7:**
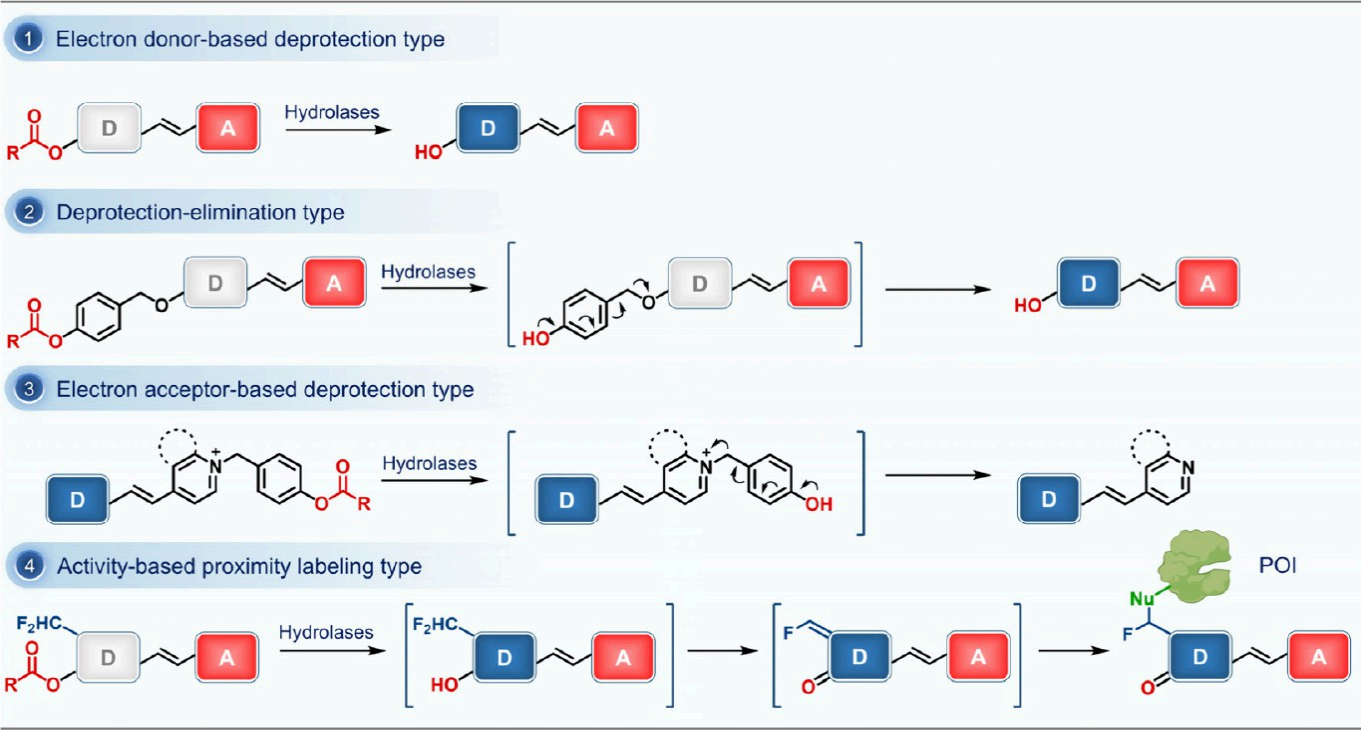
Hydrolase-activatable probe designs. (1) The RM-deprotection strategy restores fluorescence after enzymatic cleavage. (2) Upon enzymatic cleavage, a self-immolative group undergoes rapid 1,6-rearrangement elimination, emitting intense fluorescence. (3) Cascade deprotection and elimination convert quaternary ammonium salt to tertiary amine, inducing spectral changes. (4) Hydrolase-triggered proximity labeling utilizes a difluoromethyl group as a precursor to generate a reactive electrophilic QM.

#### 3.2.1 Esterases

Esterase-activatable probes can be flexibly tailored to target distinct isoforms through strategic optimization of the ester group. The two predominant carboxylesterase isoforms, CES1A and CES2A, exhibit distinct substrate preferences. CES1A favors substrates with a bulky acyl moiety and a small alcohol moiety, whereas CES2A prefers substrates with a large alcohol group and a small acyl group. Accordingly, CES1A probes usually possess an oversized fluorophore bearing a carboxyl group as the fluorescent switch (Figure 8A),^77,78,111^ while CES2A probes typically employ a small acyl group such as a chloroacetyl moiety (Figure 8B).^50,79^ Beyond substrate-mimetic approaches, covalent inhibitor-based targeted labeling strategies have emerged as powerful alternatives. A notable example is the development of a covalent probe 25 for sensing monoacylglycerol lipase (MAGL) that integrates a BODIPY fluorophore into the aromatic pharmacophore of MAGL inhibitors, enabling specific labeling of MAGL in living cells, hippocampal neuron cultures, and human-induced pluripotent stem cell (hiPSC)-derived brain organoids (Figure 8C).^55^ Radiolabeling of the analog of this probe further enabled multiscale PET imaging, revealing high MAGL expression in hippocampal and cortical regions.

**Figure 8:**
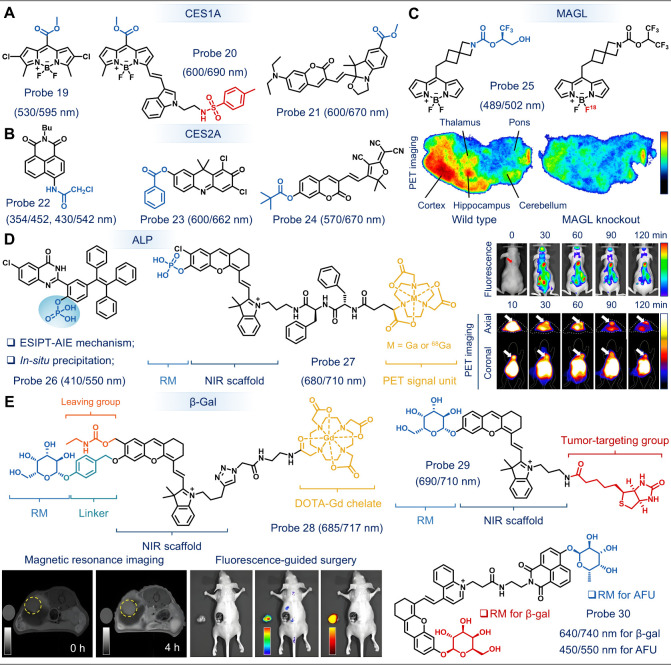
(AB) CES1A and (B) CES2A probes. (C) (left) Covalent MAGL probe exhibited highly drug-like properties and wide utility for activity-based profiling, cell imaging, and flow cytometry. (right) *In vitro* autoradiography of MAGL distribution in brain slices from wild-type and MAGL knockout mice using a dual-modality probe. Reproduced from ref 55. CC BY 4.0. (D) ALP probes 26 and 27. Probe 27 exhibited bright NIR fluorescence and rapid radioactive accumulation in the subcutaneous HeLa tumors. Reproduced from ref 82. Copyright 2021 American Chemical Society. (E) β-Gal probes 28–30. MRI revealed effective accumulation of probe 28 in ovarian tumors of live mice. After tail vein injection, probe 28 accurately delineated the ovarian tumor margins, enabling precise surgical resection. Reproduced with permission from ref 62. Copyright 2023 John Wiley and Sons.

For hydrolases metabolizing highly water-soluble substrates, such as alkaline phosphatase (ALP), enzyme-triggered precipitation or self-assembly strategies effectively counteract signal diffusion. Probe 26 released a solid-state emissive fluorophore upon ALP activation, allowing for the diffusion-resistant localization of ALP activity in living cells and osteosarcoma tissues (Figure 8D).^81^ This design overcame the limitation of signal diffusion associated with conventional soluble fluorophores, allowing precise *in situ* mapping of enzyme activity. Expanding on this concept, probe 27 underwent ALP-triggered dephosphorylation followed by *in situ* nanoparticle assembly, concurrently amplifying NIR fluorescence and radioactive signals for deep-tissue imaging of ALP-expressing tumors *in vivo*.^82^ These examples highlight two scalable strategies for designing esterase probes. First, tailoring the steric and electronic properties of the ester group to match isoform-specific substrate preferences. Second, incorporating self-immolative linkers or enzyme-triggered assembly strategies to overcome signal diffusion, particularly for hydrolases processing highly water-soluble substrates. These strategies enable precise visualization of esterase activity across biological scales from subcellular compartments to *in vivo* models.

#### 3.2.2 Glycosidase

β-Galactosidase (β-Gal), a key biomarker for cell senescence and primary ovarian cancers, is markedly upregulated in the lysosome of senescent cells. β-Gal probes are typically designed by introducing a β-D-galactose moiety as a cleavable trigger to suppress fluorescence.^112^ A self-immobilizing probe 28 combined NIR fluorescence and magnetic resonance imaging (MRI) to enable real-time visualization of β-Gal activity in living organisms (Figure 8E).^62^ Upon β-gal cleavage, an electrophilic QM intermediate was released and covalently labeled adjacent target enzymes, enhancing signal retention at the tumor site and facilitating precise surgical resection of ovarian tumors in mice. To enhance tumor-targeting, probe 29 incorporated a tumor-targeting group biotin for specific uptake, enabling dual fluorescent/photoacoustic imaging of β-gal activity in senescent tumors, with high specificity and deep-tissue penetration.^83^ Further advancing this concept, a dual-enzyme activated probe 30 enabled simultaneous and independent detection of β-gal and α-l-fucosidase (AFU) via distinct emission at 740 and 550 nm, respectively.^84^ This design significantly enhanced the accuracy of senescent cell identification and precise tracking in β-Gal-overexpressing ovarian cancer models. The evolution of β-Gal probes illustrates three complementary strategies for improving imaging fidelity: covalent signal retention to overcome diffusion, active targeting to enhance tumor specificity, and dual-enzyme activation to increase diagnostic accuracy. These probes provide a versatile toolkit for real-time detection and imaging of cellular senescence, supporting both mechanistic studies and therapeutic evaluation.

#### 3.2.3 Amidases

Amidases play critical roles in amide bond hydrolysis, contributing to lipid signaling and oxidative stress responses. Their diverse catalytic characteristics have spurred the development of tailored molecular probes. For fatty acid amide hydrolase (FAAH), which preferentially cleaves hydrophobic long-chain fatty amide substrates, probe 31 was designed by introducing arachidonic acid as a specific RM into a 7-amino-3*H*-phenoxazin-3-one scaffold (Figure 9A).^85^ This probe enabled high-throughput inhibitor screening, leading to the identification of the potent natural FAAH inhibitor neobavaisoflavone. Vanin-1 (pantetheinase), an epithelial glycosylphosphatidylinositol-anchored ectoenzyme, catalyzes the cleavage of pantetheine into the amino-thiol cysteamine and pantothenic acid. Probe 32 was constructed by linking pantothenic acid to methylene blue (MB) via a self-eliminating linker (Figure 9B).^86^ Vanin-1-mediated cleavage triggered an 85-fold fluorescence enhancement with rapid response (within 5 min), enabling sensitive detection of inflammation in mouse models. Beyond mammalian systems, amidase probes are also advancing microbiology research. A bacteria-targeting NIR probe 33 for penicillin G acylase (PGA) was designed by incorporating the phenylacetyl moiety into cyanine dyes (Figure 9C).^87^ This probe enabled visualization of biofilm regulation in *Acinetobacter baumannii* and, through its PTT and PDT properties, facilitated effective treatment of bacterial infection and wound healing. The development of amidase probes demonstrates how integrating activity-based sensing with additional functionalities extends probe utility beyond simple detection toward functional intervention.

**Figure 9:**
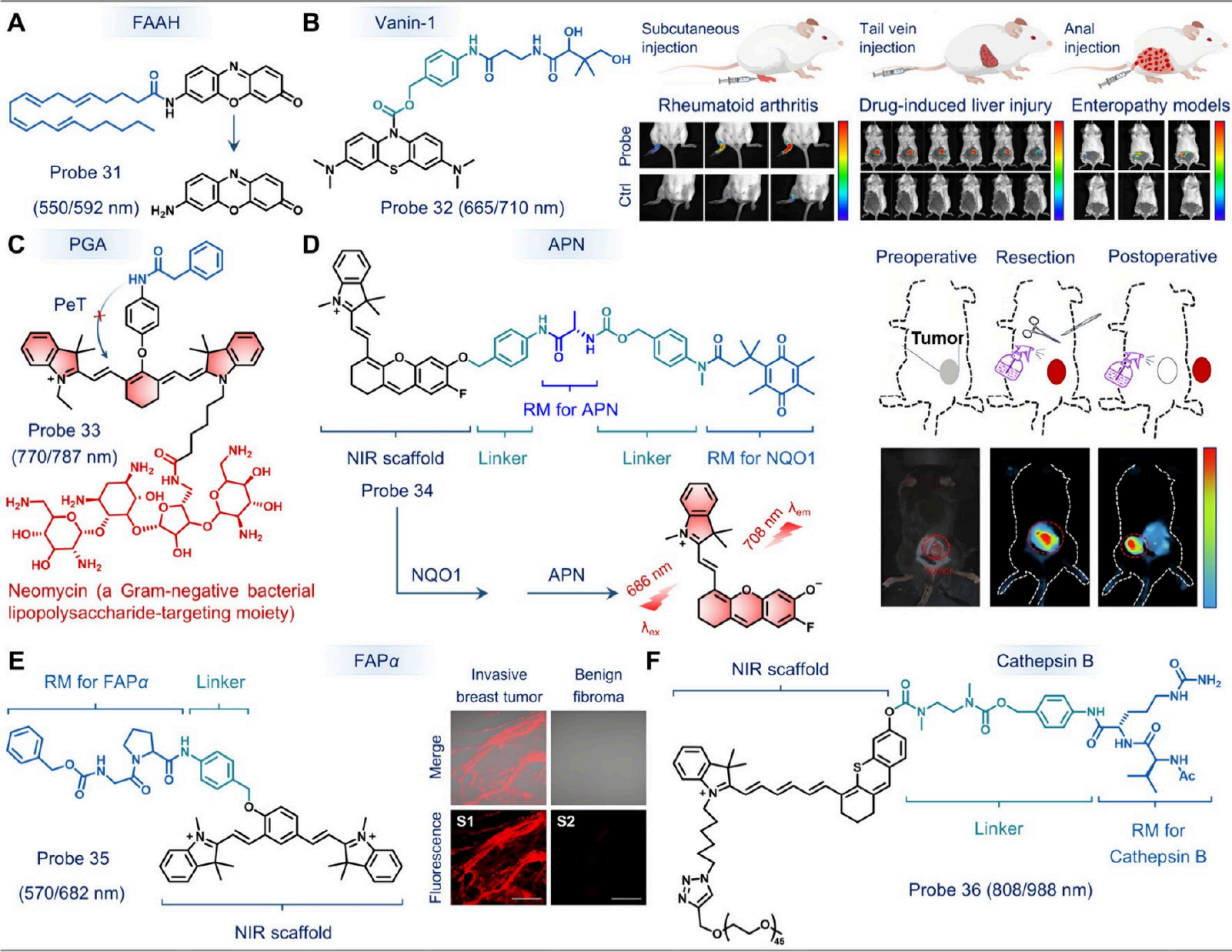
(A) Sensing mechanism of probe 31 for FAAH. (B) Probe 32 for Vanin-1 and fluorescence imaging of Vanin-1 levels in inflammatory tissues of various mouse models. Reproduced from ref 86. Copyright 2025 American Chemical Society. (C) PGA-responsive probe 33. (D) APN-responsive probe 34. Fluorescence imaging of NQO1 and APN after spraying probe 34 in the preoperative and postoperative stages. Reproduced with permission from ref 88. Copyright 2024 Wiley-VCH. (E) Probe 35 for FAPα and confocal images of human invasive breast tumors and benign fibroadenoma tissue. Reproduced with permission from ref 89. Copyright 2025 Wiley-VCH. (F) Probe 36 for cathepsin B.

#### 3.2.4 Peptidases/Proteases

Peptidases and proteases are central regulators of extracellular matrix remodeling, tumor invasion, and immune modulation. To improve tumor margin delineation, an “offense–defense integration” (ODI) strategy that leveraged the attack systems (invasive peptidase) and defense systems (reductive microenvironment) of tumors was proposed to design dual-locked probe 34 (Figure 9D).^88^ This system enabled precise discrimination between malignant and normal tissues, effectively achieving accurate intraoperative tumor resection in mouse models and clinical specimens. Similarly, a positive charge-driven optimization strategy yielded probe 35, a FAPα-activated probe with markedly enhanced sensitivity, in which two positive charges facilitated access to the enzyme’s active sites (Figure 9E).^89^ This tool enables high-resolution tumor margin delineation and assessment of invasive potential, serving as a valuable intraoperative navigation aid. Expanding into the shortwave infrared (SWIR) window, probe 36 was engineered from hemicyanine derivatives with extended polymethine bridges and sulfur substitution, achieving bathochromic shifts that improve tissue penetration and reduce scattering (Figure 9F).^90^ This cathepsin B-activatable probe enabled high-contrast, highly sensitive identification of metastatic lung lesions in unshaved mice. The development of peptidase/protease probes underscores the value of integrating multiple tumor microenvironment cues, such as enzyme activity, redox state, and tissue architecture, into a single probe design to enhance specificity. By merging advanced substrate engineering with adaptive activation mechanisms, these evolving probe systems are poised to make substantial contributions to tumor staging, intraoperative guidance, and precision therapeutic delivery.

### 3.3 Transferases

In phase II drug metabolism, transferases mediate the conjugation of functional groups (e.g., glucuronyl, sulfate, methyl, acyl, and glutathione groups) to xenobiotics or phase I metabolites, enhancing aqueous solubility and facilitating excretion (Figure 10A). Representative members include UDP-glucuronosyltransferases (UGTs), sulfotransferases (SULTs), catechol-O-methyltransferases (COMTs), *N*-acetyltransferases (NATs), and glutathione *S*-transferases (GSTs). The sensing paradigm of transferase-activatable probes relies on conjugation-induced signal modulation, which differs from hydrolase-mediated cleavage. For ICT-based or ESIPT-based probes, transferase-mediated conjugative groups directly modify the probe’s auxochrome (e.g., −OH, −NH_2_), typically passivating the electron donor or blocking proton transfer, leading to fluorescence quenching or a blue shift. Conversely, if the probe’s metabolic site is also a switch for the PeT process, its modification can inhibit the PeT pathway and restore fluorescence.^101^ Therefore, selecting an appropriate sensing mode requires a comprehensive analysis of the transferred group’s impact on the probe’s electronic structure, solubility, and molecular conformation. An often-overlooked yet critical consideration is the significant increase in hydrophilicity, which can trigger complete subcellular redistribution of the probe. Ignoring this metabolism-dependent translocation risks misinterpreting imaging results.

**Figure 10:**
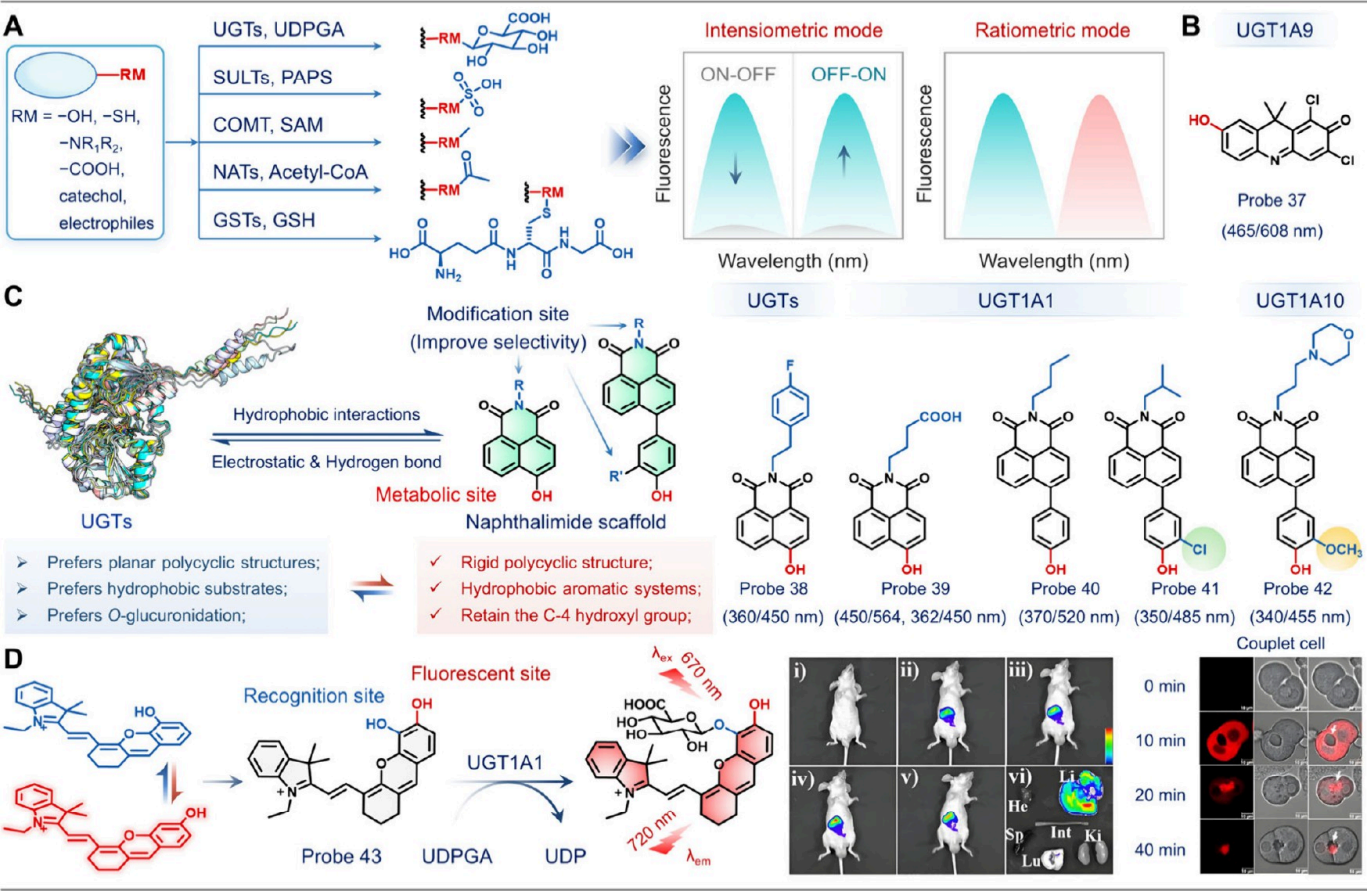
(A) Representative transferase-mediated metabolism and associated spectroscopic variations. (B) UGT1A9-specific probe 37. (C) Metabolic potential and modification strategies of 1,8-naphthalimide-based UGT fluorogenic substrates, including broad-spectrum probe 38, UGT1A1 specific probes 39–41, and UGT1A10-specific probe 42. (D) Rational construction of UGT1A1-selective probe 43 via a molecular-splicing strategy. (i–v) Fluorescence imaging of UGT1A1 after intraperitoneal administration of probe 43 for 0–30 min. (vi) *In vitro* imaging of UGT1A1 in isolated organs. Abbreviations: He, heart; Lu, lung; Gb, gallbladder; Li, liver; *K*_i_, kidney; Sp, spleen; Bl, bladder. The dynamic translocation of the NIR fluorescence signal from the cytoplasm to the bile canaliculus in hepatocyte couplet cells over 40 min, demonstrating that the glucuronide of probe 43 was actively excreted into the bile. Reproduced with permission from ref 53. Copyright 2021 John Wiley and Sons.

#### 3.3.1 UDP-glucuronosyltransferases (UGTs)

UGTs catalyze the transfer of glucuronic acid from UDP-glucuronic acid (UDPGA) to hydrophobic substrates, responsible for the metabolism of endogenous substances (e.g., bilirubin) and xenobiotics (e.g., SN-38).^113^ Among UGT subfamilies, UGT1A and UGT2B are particularly important in drug metabolism, with isoforms such as UGT1A9, UGT2B7 serving as key biomarkers for DDI assessment. Most UGT probes exploit ICT or PeT mechanisms. For ICT-based systems, glucuronidation of phenolic hydroxyl groups generally results in fluorescence quenching or a blue shift. For instance, the NIR fluorophore DDAO was an isozyme-specific substrate for UGT1A9, enabling functional monitoring in living cells and identification of environmental pollutants that disrupt its activity (Figure 10B).^91^ The naphthalimide scaffold, with its hydrophobic aromatic ring system, aligns well with UGT substrate preferences. Systematic variation of the N substituent modulates isozyme selectivity and metabolic rates (Figure 10C). The broad-spectrum probe 38, bearing a fluorobenzene moiety, was readily O-glucuronidated by 10 UGTs, exhibiting high reactivity and superb affinity.^92^ whereas replacement of the N site with butyric acid yielded probe 39 with excellent specificity but poor affinity (*K*_m_ = 126.7 μM) for UGT1A1.^93^

Building on this scaffold, a PeT-based fluorophore, 4-phenyl-1,8-naphthalimide, exhibits low background fluorescence, improved hydrophobicity, and favorable metabolic profiles. Through systematic optimization of the N substituent and phenolic group, the isoform-selective probes for UGT1A1 (probes 40 and 41) and UGT1A10 (probe 42) were devised, achieving high conversion rates and excellent isoform selectivity.^94–96^ In parallel, a “molecular-splicing” approach that integrates the optimal metabolic site with a fluorescence activation site within a hemicyanine scaffold yielded probe 43, enabling real-time visualization of UGT1A1 activity and biliary excretion *in vivo* (Figure 10D).^53^ This platform facilitated the high-throughput screening of herbal medicines, identifying kurarinone and kushenol N as potent UGT1A1 inhibitors. Despite these advances, UGT probe development remains constrained by substantial substrate promiscuity among isozymes and the scarcity of crystal structures. Deeper exploration of substrate structure-metabolism relationships, combined with computational approaches such as homology modeling and molecular docking, will accelerate the rational design of next-generation UGT-targeted tools for activity monitoring and inhibitor discovery across multiple biological scales.

#### 3.3.2 Sulfotransferases (SULTs)

SULTs catalyze the transfer of a sulfuryl moiety (−SO_3_H) from 3′-phosphoadenosine-5′-phosphosulfate (PAPS) to nucleophilic sites (e.g., phenolic, amino groups) of endogenous substances (e.g., estrogens) and xenobiotics.^114^ Advances in SULT1E1 probes illustrate a clear trajectory for molecular optimization. Structure-based virtual screening and enzymatic phenotyping initially identified *N*-butyric acid-4-hydroxy-1,8-naphthalimide as an OFF-ON probe for SULT1E1, exhibiting a 69-fold fluorescence enhancement at 450 nm and a conversion rate of 28.4% (Figure 11A).^3^ A “molecular growth” strategy that expanded the phenolic hydroxyl group while retaining the *N*-butyric acid moiety yielded probe 44, achieving a higher conversion rate (91.1%) and an improved SNR (199-fold), with intense fluorescence around 510 nm upon SULT1E1 activation. This probe enabled the monitoring of SULT1E1 activity in clinical hepatocellular carcinoma specimens and live organs, as well as high-throughput inhibitor screening. Further refinement involving the replacement of the *N*-butyric acid group with a *p*-toluenesulfonamide moiety yielded probe 45, which exhibited increased metabolic clearance and an enhanced SNR (212-fold), enabling precise sensing of SULT1E1 activity in cellular and liver preparations.^97^ The iterative optimization of the SULT1E1 probes underscores the value of structure–activity relationship studies in guiding rational design. Meanwhile, persistent challenges, such as the scarcity of suitable scaffolds and limited understanding of the substrate spectra for SULT isoforms, point to key areas for future investigation.

**Figure 11:**
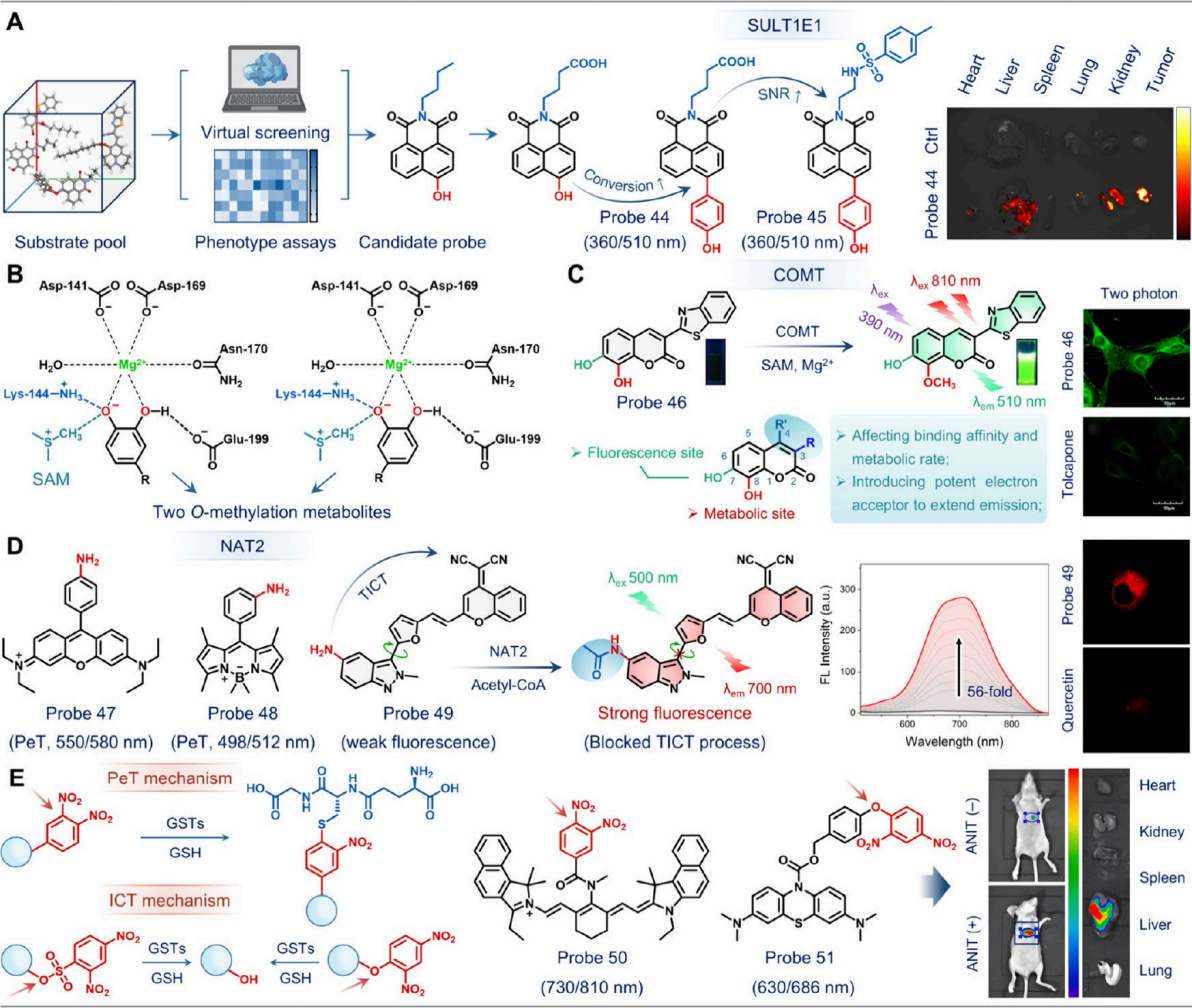
Representative transferase-activatable probes. (A) Rational engineering and performance optimization of SULT1E1 probes. Reproduced from ref 3. CC BY 4.0. (B) Binding pose of a substituted catechol substrate in the COMT active site. (C) Structure–COMT metabolism relationship of coumarin substrates. Reproduced with permission from ref 98. Copyright 2017 Wiley-VCH. (D) Chemical structures of NAT2 probes. Emission spectra of probe 49 upon NAT2 addition. Fluorescence imaging of NAT2 in HepG2 cells untreated or treated with quercetin (NAT inhibitor). Reproduced from ref 101. CC BY 4.0. (E) Responsive mechanism and chemical structures of GST probes. Functional imaging of GST levels in ANIT-induced cholestatic mouse models. Reproduced with permission from ref 103. Copyright 2022 Elsevier.

#### 3.3.3 Catechol-*O*-methyltransferases (COMTs)

COMTs catalyze the transfer of a methyl group from *S*-adenosyl-l-methionine (SAM) to a hydroxyl group of catechol substrates in the presence of Mg^2+^, playing an essential role in the metabolic inactivation of catecholamine neurotransmitters and the elimination of catechol drugs (e.g., levodopa). A longstanding challenge in COMT probe design has been the low regioselectivity of traditional catechol substrates, which typically yield two O-methylated isomers with negligible changes in fluorescence (Figure 11B).^115^ A pivotal breakthrough came with the discovery that natural 7,8-dihydroxycoumarins (e.g., 4-methyldaphnetin) can be metabolized by COMTs with high regioselectivity at the C-8 phenolic group.^116^ This specific 8-O-methylation disrupts the intrinsic fluorescence quenching induced by the catechol moiety, thereby switching on the fluorescence. On the basis of this finding, an optimized two-photon probe was developed by introducing a benzothiazole group at the C-3 position, displaying improved two-photon absorption, quantum yield, and tissue penetration (Figure 11C).^98^ This probe enabled high-throughput inhibitor screening, high-resolution imaging of endogenous COMTs in living cells and rat brain slices, and assessment of interindividual enzyme activity variations in human erythrocytes. Looking forward, the 7,8-dihydroxycoumarin scaffold offers a versatile platform for further optical optimization. By introducing a potent electron acceptor at the C-3 site, the push–pull system can be strengthened, effectively shifting the emission profiles into the NIR region and opening new frontiers for deeper-tissue imaging of COMT activity.^16^

#### 3.3.4 *N*-Acetyltransferases (NATs)

Arylamine NATs catalyze the acetylation of arylamine, arylhydrazine, arylhydrazide, and *N*-hydroxyarylamine substrates in the presence of acetyl coenzyme A. The two main isoforms, NAT1 and NAT2, with NAT2 primarily responsible for activating and deactivating arylamine and hydrazine drugs such as isoniazid. A PeT-based rhodamine probe 47 was designed to visualize NAT2 activity across diverse bacterial strains, enabling identification of the potent inhibitor kushenol C (Figure 11D).^99^ Similarly, probe 48 emitted bright fluorescence upon the addition of NAT2, allowing for the visualization of NAT2 in mycobacteria and the discovery of natural inhibitors such as isobavachalcone from Psoraleae Fructus.^100^ Distinct from conventional ICT probes, probe 49 incorporated an indazole unit into an ICT scaffold, resulting in fluorescence quenching in its native state.^101^ NAT2-mediated acetylation restricts this rotation, switching the probe to a light-up state. This innovative design that overcomes the quenching typically associated with ICT-based transferase probes and enables high-contrast imaging of NAT2 in living cells and tissues. Collectively, these probes facilitate the monitoring of NAT2 function in living systems, expanding the molecular toolkit of transferases.

#### 3.3.5 Glutathione S-Transferases

GSTs catalyze the nucleophilic addition of glutathione (GSH) to electrophilic substrates, thereby degrading toxic substances and promoting excretion. GST probes usually incorporate an electrophilic moiety that quenches fluorescence and restores fluorescence through GST-catalyzed GSH conjugation.^1^ Common reactions include the cleavage of the 2,4-dinitrobenzenesulfonate group and the nucleophilic aromatic substitution (S_N_Ar) of 3,4-dinitrobenzanilide derivatives (Figure 11E). An NIR probe integrated 3,4-dinitrobenzoic acid as a responsive moiety into a benzoheptamethine cyanine scaffold for the detection of GSTs.^102^ Upon GST-catalyzed glutathionylation via a two-step S_N_Ar addition/elimination reaction, a sulfhydryl substitution derivative was generated, which suppressed the PeT process and restored fluorescence. Probe 50 revealed elevated GST levels in pulmonary fibrosis cells, mouse models, and idiopathic pulmonary fibrosis (IPF) patients. Furthermore, the study demonstrated that combining a GST inhibitor (TLK117) with pirfenidone displayed superior therapeutic outcomes compared to pirfenidone alone in pulmonary fibrosis mouse models, highlighting GST inhibition may be a promising synergistic strategy for IPF treatment. Potential interference from abundant GSH in biological systems may affect the detection of the 2,4-dinitrobenzenesulfonate group for GSTs. By tuning the sulfonic acid group and the position of the nitro group, probe 51 was optimized using the 2,4-dinitrobenzene group as the response site.^103^ This tool successfully monitored overexpressed GSTs in α-naphthylisothiocyanate (ANIT)-induced cholestatic mice and detected the upregulation of GST activity in patients with intrahepatic cholestasis of pregnancy (ICP).

## 4 Challenges and Future Perspectives

Over the past decade, a diverse arsenal of EAFPs has been engineered to uncover the dynamic panorama of target enzymes, enabling both mechanistic elucidation and the targeted intervention of pathological processes. By conversion of enzymatic activity into detectable optical signals, these probes afford high spatiotemporal resolution from subcellular to *in vivo* levels, driving advances in biomarker discovery, intraoperative navigation, and drug screening. Despite these successes, the journey toward developing clinically translatable platforms remains challenged by scaffold innovation, sensing performance, and functional integration.

High-performance fluorophores are essential for tailoring EAFPs to various application scenarios. Ideal candidates should possess tunable photophysical properties, flexible modification sites, and favorable biocompatibility. While traditional dyes such as BODIPY and rhodamine derivatives remain reliable, their limited emission restricts deep-tissue imaging. A leading direction is to develop activatable NIR-I (700–900 nm) and NIR-II (1000–1700 nm) probes for high-contrast *in vivo* imaging.^7,117,118^ AI and density functional theory (DFT) accelerate the prediction and regulation of emissive wavelengths, enabling custom spectral design.^119^ Beyond fluorescence, integration with PA, MRI, or PET modalities overcomes the limited tissue penetration while preserving spatial resolution, offering more comprehensive readouts.^62,82^ Future expansion into MRI-active or radiolabeled scaffolds will further broaden the imaging versatility.^9,82,120^

Beyond optical performance, achieving target specificity of EAFPs in complex biological environments demands rational strategies. This begins with a deep understanding of enzyme substrate preferences, active site topology, and isoform-specific catalytic features.^31^ Structurally, self-immolative linkers can decrease steric constraints imposed on bulky fluorophores by the catalytic site, improving turnover kinetics. Dual-locked or AND-logic probes, which require concurrent enzymatic and microenvironmental inputs, offer a robust paradigm for improving biosensing accuracy in disease-specific settings.^121^ Furthermore, multiscale computational tools enable the RM to better match the topological and electronic features of the active site.^38^ For instance, ensemble docking predicts the spatial distance between the metabolic site and catalytic residue by sampling multiple receptor conformations, moving beyond single static structures.^15^ Molecular dynamics (MD) simulations offer insights into binding stability and dynamic interaction. Quantum mechanical/molecular mechanical (QM/MM) calculations can model the electronic rearrangement during the catalytic process. Notably, AI is accelerating high-affinity fragment identification and substrate-enzyme interaction prediction.^15,122^ Integrating these computational layers enables systematic engineering of EAFPs with desired attributes, substantially shortening development timelines.

Beyond molecular specificity, the spatiotemporal fidelity of the fluorescent signal, i.e., preventing spontaneous diffusion of the activated fluorophore, is equally critical for accurate imaging. Covalent labeling allows *in situ* retention, yet its always-on nature often results in poor SNR and obscures dynamic activity. To overcome this limitation, emerging strategies integrate activatable responses with *in situ* retention. Enzyme-triggered self-immobilization, utilizing acyloxymethyl ketone (AOMK) warheads, QM intermediates, or photo-cross-linking groups, achieves covalent anchoring to proximal proteins for high-fidelity mapping of enzyme activity and localization.^34^ Alternatively, noncovalent approaches, including *in situ* precipitation of activated fluorophores, enzyme-triggered self-assembly, or lipophilicity modulation, enable physical confinement without covalent modification.^50,81,82^ Collectively, these strategies substantially improve imaging precision and support long-term tracking.

The field is evolving from pure imaging tools toward integrated theranostic platforms. This trend manifests in several transformative advances. The fusion of NIR fluorescence with photoacoustic or radionuclide imaging facilitates real-time intraoperative navigation and precise tumor resection.^9,123^ “Self-reporting” therapeutic probes unite enzyme-activated diagnostics with photodynamic or photothermal ablation, forming closed-loop systems for image-guided precision therapy. Meanwhile, EAFP-driven high-throughput screening platforms directly visualize drug-induced modulation of enzyme activity, accelerating lead optimization and integrated pharmacokinetic-pharmacodynamic (PK–PD) evaluations.^4,54^ Future breakthroughs will likely hinge on deeper interdisciplinary convergence, combining innovations in organic chemistry, structural biology, computational modeling, and materials science to bridge the gap between molecular sensing and clinical intervention. Addressing the triad of scaffold innovation, sensing performance, and functional integration will be key to unlocking the full translational potential of EAFPs.

## References

[1] Jin Q., Wu J., Wu Y., Li H., Finel M., Wang D., Ge G. (2022). Optical substrates for drug-metabolizing enzymes: Recent advances
and future perspectives. Acta Pharm. Sin. B.

[2] Wu J., Guan X., Dai Z., He R., Ding X., Yang L., Ge G. (2021). Molecular
probes for human cytochrome
P450 enzymes: Recent progress and future perspectives. Coord. Chem. Rev..

[3] Niu X., Fan Y., Zhu G., Zeng H., Zhao B., Sun M., Chen L., Wu L., Tian Z., James T. D., Ge G. (2025). Rational engineering
of isoform-specific hSULT1E1 fluorogenic substrates
for functional analysis and inhibitor screening. Biosens. Bioelectron..

[4] Zhang F., Zhao B., Fan Y., Qin L., Shi J., Chen L., Xu L., Jin X., Sun M., Deng H., Zeng H., Xiao Z., Yang X., Ge G. (2025). Discovery of a novel AhR-CYP1A1 axis activator for mitigating inflammatory
diseases using an in situ functional imaging assay. Acta Pharm. Sin. B.

[5] Fan Y., Wang F., Hou F., Wei L., Zhu G., Zhao D., Hu Q., Lei T., Yang L., Wang P., Ge G. (2023). A novel TICT-based
near-infrared
fluorescent probe for light-up sensing and imaging of human serum
albumin in real samples. Chin. Chem. Lett..

[6] Kang X., Du Z., Yang S., Liang M., Liu Q., Qi J. (2024). Smart molecular
probes with controllable photophysical property for smart medicine. Smart Mol..

[7] Hu X., Yao C., Wang B., Zhang Y., Yang J., Dong Y., Li Y., Wang D., Chen X., Deng Y., Ge G., Zhou B., Luo X., Qian X., Yang Y. (2025). Near-infrared
biosensing of drug-induced cell-heterogeneous injuries with an ultrahigh
turn-on ratio. Angew. Chem., Int. Ed..

[8] Li W., Ai S., Zhu H., Lin W. (2025). Activatable second-near-infrared-window
multimodal luminogens with aggregation-induced-emission and aggregation-caused-quenching
properties for step-imaging guided tumor therapy. Nat. Commun..

[9] Yan R., Hu Y., Liu F., Wei S., Fang D., Shuhendler A. J., Liu H., Chen H. Y., Ye D. (2019). Activatable NIR fluorescence/MRI
bimodal probes for in vivo imaging by enzyme-mediated fluorogenic
reaction and self-assembly. J. Am. Chem. Soc..

[10] Chen M., Wang C., Ding Z., Wang H., Wang Y., Liu Z. (2022). A molecular logic gate for developing “AND” logic probes
and the application in hepatopathy differentiation. ACS Cent. Sci..

[11] Peng H., Wang T., Li G., Huang J., Yuan Q. (2022). Dual-locked
near-infrared fluorescent probes for precise detection of melanoma
via hydrogen peroxide-tyrosinase cascade activation. Anal. Chem..

[12] Li Y., Zuo S., Chen Y., Zhou J., Shi L., Yuan L. (2025). Enzyme-activatable
dual-locked fluorescent probe for precision imaging of cutaneous squamous
cell carcinoma. Smart Mol..

[13] Zhu H., Oh J. H., Matsuda Y., Mino T., Ishikawa M., Nakamura H., Tsujikawa M., Nonaka H., Hamachi I. (2024). Tyrosinase-based
proximity labeling in living cells and in vivo. J. Am. Chem. Soc..

[14] Liu T., Xia X., Wang R., Rong X., Su Z. H., Du J. J., Fan J. L., Peng X. J., Sun W. (2023). A Fluorescent chemosensor
for long-term tracking of cancer cell metastasis and invasion via
enzyme-activated anchoring. Adv. Funct. Mater..

[15] Zhang F., Song L., Wang R., Zhao B., Huang J., Wu L., Fan Y., Lin H., Jiang Z., Yang X., Zeng H., Yang X., James T. D., Ge G. (2025). Functional
imaging of CYP3A4 at multiple dimensions using an AI-driven high performance
fluorogenic substrate. Small.

[16] Fan Y., Wu Y., Hou J., Wang P., Peng X., Ge G. (2023). Coumarin-based
near-infrared fluorogenic probes: Recent advances, challenges and
future perspectives. Coord. Chem. Rev..

[17] Zeng Z., Liew S. S., Wei X., Pu K. (2021). Hemicyanine-based near-infrared
activatable probes for imaging and diagnosis of diseases. Angew. Chem., Int. Ed..

[18] Karton-Lifshin N., Segal E., Omer L., Portnoy M., Satchi-Fainaro R., Shabat D. (2011). A unique paradigm for
a Turn-ON near-infrared cyanine-based
probe: noninvasive intravital optical imaging of hydrogen peroxide. J. Am. Chem. Soc..

[19] Redy-Keisar O., Kisin-Finfer E., Ferber S., Satchi-Fainaro R., Shabat D. (2014). Synthesis and use of
QCy7-derived modular probes for
the detection and imaging of biologically relevant analytes. Nat. Protoc..

[20] Zhu H., Fan J., Du J., Peng X. (2016). Fluorescent probes for sensing and
imaging within specific cellular organelles. Acc. Chem. Res..

[21] Yue D., Wang M., Deng F., Yin W., Zhao H., Zhao X., Xu Z. (2018). Biomarker-targeted
fluorescent probes
for breast cancer imaging. Chin. Chem. Lett..

[22] Gao P., Pan W., Li N., Tang B. (2019). Fluorescent probes for organelle-targeted
bioactive species imaging. Chem. Sci..

[23] Singh D., Rajput D., Kanvah S. (2022). Fluorescent
probes for targeting
endoplasmic reticulum: design strategies and their applications. Chem. Commun..

[24] Zhang L. J., Huang R., Shen Y. W., Liu J., Wu Y., Jin J. M., Zhang H., Sun Y., Chen H. Z., Luan X. (2021). Enhanced anti-tumor efficacy by inhibiting
HIF-1alpha to reprogram
TAMs via core-satellite upconverting nanoparticles with curcumin mediated
photodynamic therapy. Biomater. Sci..

[25] Zhang Y., Li S., Zhang H., Xu H. (2021). Design and application of receptor-targeted
fluorescent probes based on small molecular fluorescent dyes. Bioconjugate Chem..

[26] Yang J., Li K., Hou J. T., Li L. L., Lu C. Y., Xie Y. M., Wang X., Yu X. Q. (2016). Novel tumor-specific and mitochondria-targeted
near-infrared-emission fluorescent probe for SO_2_ derivatives
in living cells. ACS Sens..

[27] Dixon L. J., Barnes M., Tang H., Pritchard M. T., Nagy L. E. (2013). Kupffer cells in the liver. Compr.
Physiol..

[28] Pujol A. M., Cuillel M., Renaudet O., Lebrun C., Charbonnier P., Cassio D., Gateau C., Dumy P., Mintz E., Delangle P. (2011). Hepatocyte targeting
and intracellular copper chelation
by a thiol-containing glycocyclopeptide. J.
Am. Chem. Soc..

[29] Jiang W. L., Li Y., Wang W. X., Zhao Y. T., Fei J., Li C. Y. (2019). A hepatocyte-targeting
near-infrared ratiometric fluorescent probe for monitoring peroxynitrite
during drug-induced hepatotoxicity and its remediation. Chem. Commun..

[30] Jiang A., Chen G., Xu J., Liu Y., Zhao G., Liu Z., Chen T., Li Y., James T. D. (2019). Ratiometric two-photon
fluorescent probe for in situ imaging of carboxylesterase (CE)-mediated
mitochondrial acidification during medication. Chem. Commun..

[31] Fan Y., Zhang F., Hao Y., Chen L., Zhou Q., Zeng H., Song Y., Guo Z., Peng X., Ge G. (2025). Fluorogenic probes for functional
imaging of endoplasmic reticulum-resident
proteins: from molecular engineering to biomedical applications. Adv. Funct. Mater..

[32] Zhang H., Fan J., Wang J., Zhang S., Dou B., Peng X. (2013). An off-on
COX-2-specific fluorescent probe: targeting the Golgi apparatus of
cancer cells. J. Am. Chem. Soc..

[33] Gurram B., Zhang S., Li M., Li H., Xie Y., Cui H., Du J., Fan J., Wang J., Peng X. (2018). Celecoxib
conjugated fluorescent probe for identification and discrimination
of cyclooxygenase-2 enzyme in cancer cells. Anal. Chem..

[34] Wu X., Wang R., Kwon N., Ma H., Yoon J. (2022). Activatable
fluorescent probes for in situ imaging of enzymes. Chem. Soc. Rev..

[35] Gardner S. H., Reinhardt C. J., Chan J. (2021). Advances in activity-based sensing
probes for isoform-selective imaging of enzymatic activity. Angew. Chem., Int. Ed..

[36] Jin Q., Ma H., Feng L., Wang P., He R., Ning J., Yang L., Ge G. (2020). Sensing cytochrome P450 1A1 activity
by a resorufin-based isoform-specific fluorescent probe. Chin. Chem. Lett..

[37] Gong Q., Yang F., Hu J., Li T., Wang P., Li X., Zhang X. (2021). Rational designed highly
sensitive NQO1-activated near-infrared
fluorescent probe combined with NQO1 substrates in vivo: An innovative
strategy for NQO1-overexpressing cancer theranostics. Eur. J. Med. Chem..

[38] Song L., Sun M., Shi J., Tian Z., Song Y., Liu H., Zhao S., Yin H., Ge G. (2023). Rational construction
of a novel bioluminescent substrate for sensing the tumor-associated
hydrolase notum. Anal. Chem..

[39] Yan C., Guo Z., Liu Y., Shi P., Tian H., Zhu W. H. (2018). A sequence-activated
AND logic dual-channel fluorescent probe for tracking programmable
drug release. Chem. Sci..

[40] Wang S., Tan W., Lang W., Qian H., Guo S., Zhu L., Ge J. (2022). Fluorogenic
and mitochondria-localizable probe enables selective
labeling and imaging of nitroreductase. Anal.
Chem..

[41] Reja S. I., Minoshima M., Hori Y., Kikuchi K. (2021). Near-infrared
fluorescent
probes: a next-generation tool for protein-labeling applications. Chem. Sci..

[42] Faucher F., Bennett J. M., Bogyo M., Lovell S. (2020). Strategies for tuning
the selectivity of chemical probes that target serine hydrolases. Cell. Chem. Biol..

[43] Miao Y., Wang Y., Chen Y., Huang Z., Lu C., Liu Y., Chen F., Wen X., Zhang J., Zhu S., Zhao P., Chen Y., Tian T., Zhang Y., Xie H., Lin J., Ye D. (2025). Pretargeted multimodal tumor imaging
by enzymatic self-immobilization labeling and bioorthogonal reaction. J. Am. Chem. Soc..

[44] Zhang Y., Lv X., Wang Y., Chen X., Zhang J., Su D. (2024). Recent advances
in self-immobilizing fluorescent probes for in vivo imaging. Smart Mol..

[45] Fang H., Peng B., Ong S. Y., Wu Q., Li L., Yao S. Q. (2021). Recent advances in activity-based probes (ABPs) and
affinity-based probes (AfBPs) for profiling of enzymes. Chem. Sci..

[46] Yadav A. K., Reinhardt C. J., Arango A. S., Huff H. C., Dong L., Malkowski M. G., Das A., Tajkhorshid E., Chan J. (2020). An activity-based sensing approach
for the detection of cyclooxygenase-2
in live cells. Angew. Chem., Int. Ed..

[47] Wu X., Wang R., Qi S., Kwon N., Han J., Kim H., Li H., Yu F., Yoon J. (2021). Rational design of
a highly selective near-infrared two-photon fluorogenic probe for
imaging orthotopic hepatocellular carcinoma chemotherapy. Angew. Chem., Int. Ed..

[48] Song Y., Zhang F., Guo J., Fan Y., Zeng H., Sun M., Qian J., Qi S., Chen Z., Jin X., Song Y., Tian T., Qian Z., Sun Y., Tian Z., Yu B., Ge G. (2025). High-efficient discovering
the potent anti-Notum agents from herbal medicines for combating glucocorticoid-induced
osteoporosis. Acta Pharm. Sin. B.

[49] Ning J., Liu T., Dong P., Wang W., Ge G., Wang B., Yu Z., Shi L., Tian X., Huo X., Feng L., Wang C., Sun C., Cui J., James T. D., Ma X. (2019). Molecular design strategy to construct the near-infrared fluorescent
probe for selectively sensing human cytochrome P450 2J2. J. Am. Chem. Soc..

[50] Fan Y., Zhang T., Song Y., Sang Z., Zeng H., Liu P., Wang P., Ge G. (2023). Rationally engineered hCES2A near-infrared
fluorogenic substrate for functional imaging and high-throughput inhibitor
screening. Anal. Chem..

[51] Song L., Sun M., Song Y., Zhang F., Zhao B., Zeng H., Shi J., Liu H., Zhao S., Tian T., Yin H., Ge G. (2024). Rationally
engineered IR-783 octanoate as an enzyme-activatable fluorogenic
tool for functional imaging of hNotum in living systems. Chin. Chem. Lett..

[52] Li Z., Wang Y. F., Zeng C., Hu L., Liang X. J. (2018). Ultrasensitive
tyrosinase-activated turn-on near-infrared fluorescent probe with
a rationally designed urea bond for selective imaging and photodamage
to melanoma cells. Anal. Chem..

[53] Tian X., Liu T., Ma Y., Gao J., Feng L., Cui J., James T. D., Ma X. (2021). A molecular-splicing strategy for
constructing a near-infrared fluorescent probe for UDP-glucuronosyltransferase
1A1. Angew. Chem., Int. Ed..

[54] Zhang F., Fan Y., Luo M., Huang J., Zhao B., Chen L., Zhu G., Xiong Y., Lin H., Xu C., Yang X., James T. D., Ge G. (2025). An optimized CYP3A4-activatable fluorogenic
sensor for in situ functional imaging and multi-dimensional inhibitor
assessment. Chem. Sci..

[55] Hentsch A., Guberman M., Radetzki S., Kaushik S., Huizenga M., He Y., Contzen J., Kuhn B., Benz J., Schippers M., Paul J., Leibrock L., Collin L., Wittwer M., Topp A., O’Hara F., Heer D., Hochstrasser R., Blaising J., von Kries J. P., Mu L., van der
Stelt M., Mergenthaler P., Lipstein N., Grether U., Nazare M. (2025). Highly specific miniaturized fluorescent monoacylglycerol
lipase probes enable translational research. J. Am. Chem. Soc..

[56] Liu R., Xu Y., Xu K., Dai Z. (2021). Current trends and key considerations
in the clinical translation of targeted fluorescent probes for intraoperative
navigation. Aggregate.

[57] Wang X., Ding Q., Groleau R. R., Wu L., Mao Y., Che F., Kotova O., Scanlan E. M., Lewis S. E., Li P., Tang B., James T. D., Gunnlaugsson T. (2024). Fluorescent
probes for disease diagnosis. Chem. Rev..

[58] Zhang Y., Chen X., Yuan Q., Bian Y., Li M., Wang Y., Gao X., Su D. (2021). Enzyme-activated near-infrared
fluorogenic probe with high-efficiency intrahepatic targeting ability
for visualization of drug-induced liver injury. Chem. Sci..

[59] Ding W., Jia M., Yao S., Liu Z., Xu F., Li M., Wang C., He W., Chen Y., Guo Z. (2025). An *N*-alkylpyridinium-substituted cyanine platform for constructing
renal-clearable near-infrared fluorogenic probes. J. Am. Chem. Soc..

[60] Zeng C., Tan Y., Sun L., Long Y., Zeng F., Wu S. (2023). Renal-clearable
probe with water solubility and photostability for biomarker-activatable
detection of acute kidney injuries via NIR-II fluorescence and optoacoustic
imaging. ACS Appl. Mater. Interfaces.

[61] Kalgutkar A. S. (2020). Designing
around Structural Alerts in Drug Discovery. J. Med. Chem..

[62] Yu Q., Zhang L., Jiang M., Xiao L., Xiang Y., Wang R., Liu Z., Zhou R., Yang M., Li C., Liu M., Zhou X., Chen S. (2023). An NIR fluorescence
turn-on and MRl bimodal probe for concurrent real-time in vivo sensing
and labeling of β-galactosidase. Angew.
Chem., Int. Ed..

[63] Dai Z. R., Ge G. B., Feng L., Ning J., Hu L. H., Jin Q., Wang D. D., Lv X., Dou T. Y., Cui J. N., Yang L. (2015). A highly selective ratiometric two-photon fluorescent probe for human
cytochrome P450 1A. J. Am. Chem. Soc..

[64] Ning J., Tian Z., Wang J., Yan F., Shi C., Zhang S., Feng L., Shu X., Cui J., James T. D., Ma X. (2024). Rational Molecular Design of a Fluorescent
Probe for Selectively Sensing Human Cytochrome P450 2D6. Angew. Chem., Int. Ed..

[65] Ning J., Wang W., Ge G., Chu P., Long F., Yang Y., Peng Y., Feng L., Ma X., James T. D. (2019). Target enzyme-activated two-photon fluorescent probes:
a case study of CYP3A4 using a two-dimensional design strategy. Angew. Chem., Int. Ed..

[66] He R. J., Tian Z. H., Huang J., Sun M. R., Wei F., Li C. Y., Zeng H. R., Zhang F., Guan X. Q., Feng Y., Meng X. M., Yang H., Ge G. B. (2023). Rationally
engineered CYP3A4 fluorogenic substrates for functional imaging analysis
and drug-drug interaction studies. J. Med. Chem..

[67] Zhang C., Fang H., Du W., Zhang D., Qu Y., Tang F., Ding A., Huang K., Peng B., Li L., Huang W. (2023). Ultrafast detection of monoamine oxidase A in live
cells and clinical glioma tissues using an affinity binding-based
two-photon fluorogenic probe. Angew. Chem.,
Int. Ed..

[68] Sun M., Huang Y., Sun X., Fu L., Wang L., Wang X., Wang X., Chen L. (2024). Evaluation of monoamine
oxidase B fluctuation in liver fibrosis cell and mice models via a
specificity fluorescent probe. Sens. Actuators,
B.

[69] Lee J., Kim H. S., Jangili P., Kang H. G., Sharma A., Kim J. S. (2021). Fluorescent probe
for monitoring hydrogen peroxide
in COX-2-positive cancer cells. ACS Appl. Bio
Mater..

[70] Zhou L., Hu C., Huang H., Ge H., Zhang Z., Cheng W., Liu H., Yang R. (2025). Precision screening and surgical resection of pan-cancer
using a tandem-locked NIR-II fluorescent probe with optimized activation
efficiency. Angew. Chem., Int. Ed..

[71] Zhang S., Liu X., Jiang B. P., Ji S. C., Chen H., Shen X. C. (2025). Dual ratiometric
single-molecule theranostic probes for photothermal therapy and real-time
quantitative evaluation of therapeutic efficacy in vivo. Anal. Chem..

[72] Zhang Y., Chen X., Yuan Q., Bian Y., Li M., Su D., Gao X. (2022). A high-performance
enzyme-activated near-infrared probe
for the sensing and tracking of tumor-related NQO1 in cells and in
vivo. Sens. Actuators, B.

[73] Peng B., Chen G., Li Y., Zhang H., Shen J., Hou J. T., Li Z. (2022). NQO-1 enzyme-activated
NIR theranostic
agent for pancreatic cancer. Anal. Chem..

[74] Michel L., Auvray M., Askenatzis L., Badet-Denisot M. A., Bignon J., Durand P., Mahuteau-Betzer F., Chevalier A. (2024). Visualization of an endogenous mitochondrial azoreductase
activity under normoxic conditions using a naphthalimide azo-based
fluorogenic probe. Anal. Chem..

[75] Yuan J., Zhou Q. H., Xu S., Zuo Q. P., Li W., Zhang X. X., Ren T. B., Yuan L., Zhang X. B. (2022). Enhancing
the release efficiency of a molecular chemotherapeutic prodrug by
photodynamic therapy. Angew. Chem., Int. Ed..

[76] Tian Z., Ding L., Li K., Song Y., Dou T., Hou J., Tian X., Feng L., Ge G., Cui J. (2019). Rational Design
of a Long-Wavelength Fluorescent Probe for Highly Selective Sensing
of Carboxylesterase 1 in Living Systems. Anal.
Chem..

[77] Han C., Zhao X., Huo X., Yu Z., Wang C., Feng L., Cui J., Tian X., Ma X. (2023). Rational design
of a NIR fluorescent probe for carboxylesterase 1 detection during
endoplasmic reticulum stress and drug-induced acute liver injury. Chem. Commun..

[78] Zhao X., Tian M., Wang Y., Yang F., Liang G., Tian X., Feng L., Cui J. (2023). A near-infrared fluorescent
probe based on a hemi-cyanine skeleton for detecting CES1 activity
and evaluating pesticide toxicity. J. Mater.
Chem. B.

[79] Jin Q., Feng L., Wang D. D., Dai Z. R., Wang P., Zou L. W., Liu Z. H., Wang J. Y., Yu Y., Ge G. B., Cui J. N., Yang L. (2015). A two-photon ratiometric
fluorescent probe for imaging carboxylesterase 2 in living cells and
tissues. ACS Appl. Mater. Interfaces.

[80] Jin Q., Feng L., Wang D. D., Wu J. J., Hou J., Dai Z.-R., Sun S. G., Wang J. Y., Ge G.-B., Cui J. N., Yang L. (2016). A highly selective near-infrared
fluorescent probe for carboxylesterase 2 and its bioimaging applications
in living cells and animals. Biosens. Bioelectron..

[81] Liu H. W., Li K., Hu X. X., Zhu L., Rong Q., Liu Y., Zhang X. B., Hasserodt J., Qu F. L., Tan W. (2017). In situ localization
of enzyme activity in live cells by a molecular probe releasing a
precipitating fluorochrome. Angew. Chem., Int.
Ed..

[82] Hu Y., Miao Y., Zhang J., Chen Y., Qiu L., Lin J., Ye D. (2021). Alkaline phosphatase enabled fluorogenic reaction and
in situ coassembly of near-infrared and radioactive nanoparticles
for in vivo imaging. Nano Lett..

[83] Wu F., Liu J., Tao M., Wang M., Ren X., Hai Z. (2023). β-Galactosidase-activatable
fluorescent and photoacoustic imaging of tumor senescence. Anal. Chem..

[84] Luo X., Hu E., Deng F., Zhang C., Xian Y. (2025). A dual-enzyme activated
fluorescent probe for precise identification of tumor senescence. Chem. Sci..

[85] Tian X., Liu T., Li L., Shao B., Yao D., Feng L., Cui J., James T. D., Ma X. (2020). Visual high-throughput screening
for developing a fatty acid amide hydrolase natural inhibitor based
on an enzyme-activated fluorescent probe. Anal.
Chem..

[86] Dai D., Zhang Z., Ma M., Li J., Zhang S., Ma P., Song D. (2025). Vanin-1-activated fluorescent probe for real-time in
vivo imaging of inflammatory responses across multiple tissue types. Anal. Chem..

[87] Liu Y., Zhang L., Liu K., Wu L.-L., Hu H.-Y. (2024). Penicillin
G acylase-responsive near-infrared fluorescent probe: unravelling
biofilm regulation and combating bacterial infections. Chin. Chem. Lett..

[88] Shen Y., Li W., Zhou Z., Xu J., Li Y., Li H., Zheng X., Liu S., Zhang X. B., Yuan L. (2024). Dual-locked
fluorescent probes activated by aminopeptidase N and the tumor redox
environment for high-precision imaging of tumor boundaries. Angew. Chem., Int. Ed..

[89] Feng Y., Xie C., Ren T. B., Xu S., Huang S., Liu S. L., Yuan L., Huan S. Y., Zhang X. B. (2025). A General strategy
to develop highly sensitive FAPα fluorescent probes for invasive
cancer detection. Angew. Chem., Int. Ed..

[90] Yang H., Li D., Wu J., Pu K. (2025). Shortwave infrared hemicyanine-6
for cancer-activated and shaving-free preclinical imaging of lung
metastasis. J. Am. Chem. Soc..

[91] Miao Y. S., Wang J. Y., Zhuang R. R., Huo X. K., Yi Z. C., Sun X. N., Yu Z. L., Tian X. G., Ning J., Feng L., Ma X. C., Lv X. (2024). A high-affinity fluorescent
probe for human uridine-disphosphate glucuronosyltransferase 1A9 function
monitoring under environmental pollutant exposure. J. Hazard. Mater..

[92] Zhou Q. H., Lv X., Tian Z. H., Finel M., Feng L., Huo P. C., Zhu Y. D., Lu Y., Hou J., Ge G. B. (2021). A fluorescence-based
microplate assay for high-throughput screening and evaluation of human
UGT inhibitors. Anal. Chim. Acta.

[93] Lv X., Ge G. B., Feng L., Troberg J., Hu L. H., Hou J., Cheng H. L., Wang P., Liu Z. M., Finel M., Cui J. N., Yang L. (2015). An optimized ratiometric fluorescent
probe for sensing human UDP-glucuronosyltransferase 1A1 and its biological
applications. Biosens. Bioelectron..

[94] Lv X., Feng L., Ai C. Z., Hou J., Wang P., Zou L. W., Cheng J., Ge G. B., Cui J. N., Yang L. (2017). A practical and high-affinity fluorescent
probe for uridine diphosphate
glucuronosyltransferase 1A1: a good surrogate for bilirubin. J. Med. Chem..

[95] Zhai X. F., Yi Y., Yu R., Kuang Y., Shaker S., Su H. F., Ye G., Liu C. R., Qiao X., Liang L., Ye M. (2022). Rational design
of a highly selective UGT1A1 probe and its application in drug discovery. Sens. Actuators, B.

[96] Zhai X. F., Fan J. J., Yi Y., Zhang M., Yuan X., Qiao X., Liang L., Ye M. (2023). Collaborative modification
strategy to develop a highly selective fluorescent probe for human
UDP-glucuronosyltransferase 1A10. Chem. Eng.
J..

[97] Niu X., Fan Y., Chen L., Deng Y., Hao Y., Zhu G., Wang L., Zhou Q., Zhu G., Ge G. (2026). Rational engineering
of an isoform-specific and sensitive turn-on estrogen sulfotransferase-activatable
fluorescent probe for functional sensing and drug discovery. Chin. Chem. Lett..

[98] Wang P., Xia Y. L., Zou L. W., Qian X. K., Dou T. Y., Jin Q., Li S. Y., Yu Y., Wang D. D., Luo Q., Ge G. B., Yang L. (2017). An optimized two-photon fluorescent
probe for biological sensing and imaging of catechol-*o*-methyltransferase. Chem.—Eur. J..

[99] Jin Y., Tian Z., Tian X., Feng L., Cui J., Ma X. (2019). A highly selective
fluorescent probe for real-time imaging of bacterial
NAT2 and high-throughput screening of natural inhibitors for tuberculosis
therapy. Mater. Chem. Front..

[100] Yan F., Tian Z., Yang Y., Tian X., Han X., Feng L., Cui J., Ma X. (2022). Fluorescence-based
visual analysis and inhibitor screening of Arylamine *N*-acetyltransferase 2, a key enzyme for tuberculosis. Sens. Actuators, B.

[101] Yan C., Guo Z., Chi W., Fu W., Abedi S. A. A., Liu X., Tian H., Zhu W. H. (2021). Fluorescence umpolung
enables light-up sensing of N-acetyltransferases and nerve agents. Nat. Commun..

[102] He N., Bai S., Huang Y., Xing Y., Chen L., Yu F., Lv C. (2019). Evaluation of glutathione *S*-transferase
inhibition effects on idiopathic pulmonary fibrosis therapy with a
near-infrared fluorescent probe in cell and mice models. Anal. Chem..

[103] Wen Y., Long Z., Bai X., Huo F., Yin C. (2022). Specific fluorescence
release based on synergistic activation of enzymes and position-dependent
of electrophilic groups to diagnose intrahepatic cholestasis of pregnancy. Chem. Eng. J..

[104] Gröer C., Busch D., Patrzyk M., Beyer K., Busemann A., Heidecke C. D., Drozdzik M., Siegmund W., Oswald S. (2014). Absolute protein
quantification of clinically relevant
cytochrome P450 enzymes and UDP-glucuronosyltransferases by mass spectrometry-based
targeted proteomics. J. Pharm. Biomed. Anal..

[105] Xue T., Dai Y., Zhang X., Cheng Y., Gu X., Ji H., Misal S., Qi Z. (2019). Ultrasensitive near-infrared fluorescent
probe with large stokes shift for real-time tracing of CYP1A1 in living
cells and zebrafish model. Sens. Actuators,
B.

[106] Dai Z. R., Feng L., Jin Q., Cheng H., Li Y., Ning J., Yu Y., Ge G. B., Cui J. N., Yang L. (2017). A practical strategy
to design and develop an isoform-specific fluorescent
probe for a target enzyme: CYP1A1 as a case study. Chem. Sci..

[107] Youdim M. B., Bakhle Y. S. (2006). Monoamine oxidase:
isoforms and inhibitors
in Parkinson’s disease and depressive illness. Br. J. Pharmacol..

[108] Wang B., Fan J., Wang X., Zhu H., Wang J., Mu H., Peng X. (2015). A Nile blue based infrared
fluorescent probe: imaging tumors that over-express cyclooxygenase-2. Chem. Commun..

[109] Oh E. T., Kim J. W., Kim J. M., Kim S. J., Lee J. S., Hong S. S., Goodwin J., Ruthenborg R. J., Jung M. G., Lee H. J., Lee C. H., Park E. S., Kim C., Park H. J. (2016). NQO1 inhibits proteasome-mediated
degradation of HIF-1α. Nat. Commun..

[110] Zeng S., Guo Z., Hao Y., Kafuti Y. S., Yang Z., Yao Q., Wang J., Peng X., Li H. (2024). Tumor-microenvironment-activatable
organic phototheranostic agents
for cancer therapy. Coord. Chem. Rev..

[111] Tian X., Yan F., Zheng J., Cui X., Feng L., Li S., Jin L., James T. D., Ma X. (2019). Endoplasmic reticulum targeting ratiometric fluorescent probe for
carboxylesterase 2 detection in drug-induced acute liver injury. Anal. Chem..

[112] Yao Y., Zhang Y., Yan C., Zhu W. H., Guo Z. (2021). Enzyme-activatable
fluorescent probes for β-galactosidase: from design to biological
applications. Chem. Sci..

[113] Lv X., Xia Y., Finel M., Wu J., Ge G., Yang L. (2019). Recent progress and challenges in
screening and characterization
of UGT1A1 inhibitors. Acta Pharm. Sin. B.

[114] Niu X., Fan Y., Zou L., Ge G. (2024). A novel fluorescence-based
microplate assay for high-throughput screening of hSULT1As inhibitors. Biosensors (Basel).

[115] Wang F. Y., Wang P., Zhao D. F., Gonzalez F. J., Fan Y. F., Xia Y. L., Ge G. B., Yang L. (2021). Analytical
methodologies for sensing catechol-*O*-methyltransferase
activity and their applications. J. Pharm. Anal..

[116] Qian X. K., Wang P., Xia Y. L., Dou T. Y., Jin Q., Wang D. D., Hao D. C., Bi X. L., Ge G. B., Yang L. (2016). A highly selective fluorescent probe for sensing activities of catechol-*O*-methyltransferase in complex biological samples. Sens. Actuators, B.

[117] Qin Z., Zhao X., Xie Y., Zhou J., Ren T. B., Yuan L. (2026). Developing enzyme activatable second
near-infrared fluorescent probes
for high-fidelity disease diagnosis in vivo. CCS Chem..

[118] Wang J., Liu Q., Li Y., Pang Y. (2024). An environmentally
sensitive zinc-selective two-photon NIR fluorescent turn-on probe
and zinc sensing in stroke. J. Pharm. Anal..

[119] Ma G., Ding Q., Zhang Y., Zeng X., Zhu K., Chen H., Zhang W., Wang Q., Huang S., Gong P., Xu Z., Hong X. (2025). Enhancing fluorescent
probe design through multilayer interaction convolutional networks:
advancing biosensing and bioimaging precision. Chem. Sci..

[120] Kang N. Y., Lee J. Y., Lee S. H., Song I. H., Hwang Y. H., Kim M. J., Phue W. H., Agrawalla B. K., Wan S. Y. D., Lalic J., Park S. J., Kim J. J., Kwon H. Y., Im S. H., Bae M. A., Ahn J. H., Lim C. S., Teo A. K. K., Park S., Kim S. E., Lee B. C., Lee D. Y., Chang Y. T. (2020). Multimodal imaging
probe development for pancreatic beta cells: from fluorescence to
PET. J. Am. Chem. Soc..

[121] Cheng P., Pu K. (2024). Enzyme-responsive,
multi-lock optical
probes for molecular imaging and disease theranostics. Chem. Soc. Rev..

[122] Sang Z., Zhang Y., Fan Y., Luan C., Liu Z., Zhang Q., Zeng H., Song Y., Huang S., Ge G. (2025). AI-driven discovery
of highly specific and efficacious hCES2A inhibitors
for ameliorating irinotecan-triggered gut toxicity. J. Med. Chem..

[123] Li H., Yao Q., Sun W., Shao K., Lu Y., Chung J., Kim D., Fan J., Long S., Du J., Li Y., Wang J., Yoon J., Peng X. (2020). Aminopeptidase
N activatable fluorescent probe for tracking metastatic cancer and
image-guided surgery via in situ spraying. J.
Am. Chem. Soc..

